# Knockdown of *ghAlba_4* and *ghAlba_5* Proteins in Cotton Inhibits Root Growth and Increases Sensitivity to Drought and Salt Stresses

**DOI:** 10.3389/fpls.2019.01292

**Published:** 2019-10-15

**Authors:** Richard Odongo Magwanga, Joy Nyangasi Kirungu, Pu Lu, Xiaoyan Cai, Yanchao Xu, Xingxing Wang, Zhongli Zhou, Yuqing Hou, Stephen Gaya Agong, Kunbo Wang, Fang Liu

**Affiliations:** ^1^Chinese Academy of Agricultural Science (ICR, CAAS) /State Key Laboratory of Cotton Biology, Institute of Cotton Research, Anyang, China; ^2^School of Biological and Physical Sciences (SBPS), Jaramogi Oginga Odinga University of Science and Technology (JOOUST), Bondo, Kenya

**Keywords:** cotton *Alba* genes, miRNAs, *cis*-regulatory elements, VIGS and wild cotton, virus-induced gene silencing (VIGS), water deficit and salt stresses, oxidant and antioxidant enzymes

## Abstract

We found 33, 17, and 20 *Alba* genes in *Gossypium hirsutum*, *Gossypium arboretum*, and *Gossypium raimondii*, respectively. The Alba protein lengths ranged from 62 to 312 aa, the molecular weight (MW) from 7.003 to 34.55 kDa, grand average hydropathy values of −1.012 to 0.609 and isoelectric (*pI*) values of −3 to 11. Moreover, miRNAs such as gra-miR8770 targeted four genes, gra-miR8752 and gra-miR8666 targeted three genes, and each and gra-miR8657 a, b, c, d, e targeted 10 genes each, while the rests targeted 1 to 2 genes each. Similarly, various *cis*-regulatory elements were detected with significant roles in enhancing abiotic stress tolerance, such as CBFHV (RYCGAC) with a role in cold stress acclimation among others. Two genes, *Gh_D01G0884* and *Gh_D01G0922*, were found to be highly induced under water deficit and salt stress conditions. Through virus-induced gene silencing (VIGS), the VIGS cotton plants were found to be highly susceptible to both water deficit and salt stresses; the VIGS plants exhibited a significant reduction in root growth, low cell membrane stability (CMS), saturated leaf weight (SLW), chlorophyll content levels, and higher excised leaf water loss (ELWL). Furthermore, the stress-responsive genes and ROS scavenging enzymes were significantly reduced in the VIGS plants compared to either the wild type (WT) and or the positively controlled plants. The VIGS plants registered higher concentration levels of hydrogen peroxide and malondialdehyde, with significantly lower levels of the various antioxidants evaluated an indication that the VIGS plants were highly affected by salt and drought stresses. This result provides a key foundation for future exploration of the Alba proteins in relation to abiotic stress.

## Introduction

Alba family proteins are mainly referred to as basic, small, and dimeric nucleic acid-binding proteins and are mainly distributed in a number of eukaryotes and the archaeal organisms (Subota et al., 2011). The Alba protein family is integral in the organization and regulation of the euryarchea genome possessing histone and as well as the crenarchea, with no histone (Reeve, 2003). The Alba proteins have a distinctive property in the regulation and organization of the organism’s genomes through acetylation and deacetylation (Goyal et al., 2012). The Alba protein binding has a very high affinity towards double-strand deoxyribonucleic acid (DNA), thus named as acetylation lower binding affinity (*Alba*) (Crnigoj et al., 2011). In *Sulfolobus solfatataricus*, a species of thermophilic archaeon, Alba proteins have been found to reversibly acetylated at lysine 6 (*Lys16*) by a homologous protein acetyltransferase (Pat) and deacetylated by a sirtuin family deacetylase (*Sir2*) (Starai and Escalante-Semerena, 2004). Apart from the acetylation at the N-terminal of the lysine residues, the Alba proteins also contain arginine–glycine–glycine (*RGG*) repeat at the C-terminal, which are important mediators of protein:RNA, and protein: protein interactions resulting in the formation of the membraneless ribonucleoprotein granules (Chong et al., 2018). The RGG domain, are closely spaced arginine–glycine–glycine repeats, it is a DNA and RNA-binding domain in various nucleic acid-binding proteins (Arribas-Layton et al., 2016). The RGG repeats affinity for the RNA is regulated by the methylation of the arginine various RGG-box proteins (Aravind et al., 2003). Structural analysis of the Alba proteins reveals the homodimer (dimeric) nature of the proteins (Bell et al., 2002).

Alba family proteins have been found to bind the DNA with no sequence specificity (Aravind et al., 2003; Kumar Verma et al., 2018). Moreover, the Alba proteins also do interact with diverse kinds of ribonucleic acid (RNA) and in addition to a number of ribonucleo-protein complexes, such as ribosomal ribonucleic acid (rRNA) and messenger ribonucleic acid (mRNA) (Xue et al., 2000). The Alba proteins have also been found to associate with both DNA and RNA (Liu et al., 2012). The Alba protein, *SshAlba* or *Ssh10b* isolated from *Sulfolobus shibatae*, has been found to bind well with double-stranded deoxyribonucleic acid and even the single-stranded deoxyribonucleic acid but do prefer the ribonucleic acids as the physiological substrates and RNA (Guo et al., 2003). In the recent past, studies have shown that *SshAlba* interacts with double-stranded ribonucleic acid (dsRNA), leading to the destabilization of the secondary structure of the RNA (Guo et al., 2014). Plants being sessile have evolved a number of survival strategies, one of which is the evolution of a diverse number of the stress-responsive genes (Nakashima and Yamaguchi-Shinozaki, 2006). The plant’s response to abiotic stress factors through molecular mechanism has been considered as the most complex mechanism, being based on the inductions and regulation of transcriptional activity of stress-related genes (Shinozaki and Yamaguchi-Shinozaki, 2007). Plants acquire tolerance to various abiotic stress factors through metabolism reprogramming and gene expression, and in turn gaining a balance among all the plant’s faculties which are necessary for plant performance (Muñoz et al., 2016). Several plants transcription factors have been found to be integral in enhancing tolerance in plants to various environmental stress factors, but it has been shown that despite the overexpression of these genes, their overexpression is not sufficient to boost plants tolerance levels toward various abiotic factors, being additional post-translational modifications may be needed—for instance, the dehydration-responsive element-binding protein 2 (*DREB2*) (Thirumalaikumar et al., 2018). Phosphorylation is a vital process in protein post-translational modifications, which affects the protein conformation, stability, and localization (Kemp, 2018). Phosphorylation functions in a number of biological processes; it translates external stimuli which in turn illicit specific response by the cell (Boudsocq, 2005). Similarly, protein degradation has also been found to play a significant role in enhancing plant stress tolerance; this occurs through ubiquitination, which refers to the covalent addition of the small protein ubiquitin to selected target proteins (Weissman, 2001).

Alba proteins are predominantly abundant in eukaryotes, more specifically in plants and protozoan (Aravind et al., 2003). Functional analysis of the Alba proteins has been done in rice (Kumar Verma et al., 2018) and other protozoans such as *Trypanosoma brucei*, *Leishmania infantum*, *Toxoplasma gondii*, and *Plasmodium* *falciparum* (Wardleworth et al., 2002). *OsAlba1*, an *Alba* gene, isolated from *Oryza sativa*, of indica species, was up-regulated by water deficit condition; it has also been found to complement yeast VIGSs, lacking the *Pop6* gene, thereby enhancing their tolerance to dehydration (Verma et al., 2014). Rice is a water plant, upregulation of the dehydration-responsive nuclear protein.

*OsAlba1* under water deficit condition indicated the integral role played by the Alba proteins in plants in enhancing their tolerance to various environmental stress factors (Choudhary et al., 2009). In addition, two Alba protein domains, *LiAlba1* and *LiAlba3*, with a molecular weight of 13 kDa and 30 kDa, respectively, to interact with each other, thus do associate with RNA-binding proteins, ribosomal units, and translation factors (Dupé et al., 2015). Two Alba homologs, *TgAlba1* and *TgAlba2* isolated from *T. gondii*, are dual in relation to subcellular localization, found both in the nucleus and cytoplasm, but are predominantly cytoplasmic proteins, and their presence in the two cellular structures shows their integral role in both nucleus and cytoplasm (Olguin-Lamas et al., 2011). The gene silencing of *TgAlbas* revealed in regulating response to stress and differentiation, *TgAlbas 1* and *TgAlbas 2*, are associated with a high number of proteins, such as the RNA-binding proteins (Gissot et al., 2013).

Being an important commercial crop, cotton production is threatened by various abiotic stress factors such as drought, salinity, and cold among others. No studies have been done elucidating the role of the Alba proteins in relation to their stress response, despite the significant contribution of the Alba proteins in eukaryotic organisms. The completion and sequencing of the three cotton species, *Gossypium arboreum* (Li et al., 2014), *Gossypium raimondii* (Wang et al., 2012), and *Gossypium hirsutum* (Li et al., 2015), have provided the needed materials for molecular studies in cotton. Therefore, in this research work, we carried out genome-wide identification of the Alba proteins in cotton, by determining the number, their distribution, gene structure, phylogenetic relationship, their expression levels, and further carried the functional analysis of two key *Alba* genes through virus-induced gene silencing (VIGS). These results will provide new insights into the biological relevance of the proteins encoded by the *Alba* genes in plants, and their future use in developing a more water-deficit and salt stress–resilient cotton genotypes.

## Materials and Methods

### Alba Protein Identification, Sequence Analysis, Phylogenetic Tree Analysis, and Subcellular Localization Prediction

The whole sequences for the Alba proteins in *G. hirsutum*, *G. arboreum*, and *G. raimondii* were retrieved from cotton research institute (http://mascotton.njau.edu.cn), Beijing genome database (https://www.bgi.com/),and phytozome 12 (http://www.phytozome.net/), respectively. The conserved domain of Alba proteins, PF01918, was downloaded from Pfam protein families’ database (http://pfam.xfam.org). The HMM profile of the Alba proteins were submitted to HMMER search (http://hmmer.janelia.org/) against *G. hirsutum*, *G. raimondii*, and *G. arboreum* protein sequences. The amino acid sequences were analyzed in order to determine the Alba domain using online tools: the NCBI Conserved Domain Database (Marchler-Bauer et al., 2017), the Simple Modular Architecture Research Tool (http://smart.embl-heidelberg.de/), and the ScanProsite tool (http://prosite.expasy.org/scanprosite/). The Alba proteins for *G. hirsutum* AD genome, *G. arboreum* of A genome, and *G. raimondii* of the D genome [together with the whole sequences for *Arabidopsis thaliana* obtained from TAIR (http://www.arabidopsis.org/)], *O. sativa* obtained from http://rice.plantbiology.msu.edu/index.shtml , *Theobroma cacao*, *Sorghum bicolor*, *Glycine max*, and *Populus trichocarpa* were all downloaded from Phytozome v12.0 (https://phytozome.jgi.doe.gov/pz/portal.html) and were used to investigate the evolutionary relationships of the Alba proteins in plants (**Table S1**). The multiple sequence alignments of all the Alba proteins were carried out by Clustal Omega, MEGA 7.0 software, using an algorithm with 1,000 bootstraps, using complete deletion of site coverage for gaps and missing data as previously outlined in the analysis of the cotton LEA proteins (Magwanga et al., 2018). An online program, ExPASy Server tool (http://www.web.xpasy.org/compute_pi/), was applied in the investigation of the physiochemical properties of all the Alba proteins obtained for the three cotton species. Finally, the subcellular localization predictions were carried out for all the three cotton species Alba proteins, through an online tool, WoLF PSORT (https://www.wolfpsort.hgc.jp/), and the results obtained were later validated by other two online tools, the Protein Prowler Subcellular Localisation Predictor version 1.2 (http://www.bioinf.scmb.uq.edu.au/pprowler_webapp_1-2/) and TargetP1.1 server (http://www.cbs.dtu.dk/services/TargetP/).

### Chromosome Location, Sequence Analysis, and Structural Analysis of the *Alba* Genes in Cotton

The information for the Alba protein sequences, genomic sequences, cDNA sequences, and chromosomal positions was retrieved from phytozome (www.phytozome.net) for *G. raimondii* and cotton functional database (https://cottonfgd.org) for *G. hirsutum* and *G. arboreum*. The genomic sequences, the coding sequences (CDS), and Newick structure for each of the cotton species protein sequence analyses were submitted to an online tool, Gene Structure Displayer Server (http://gsds.cbi.pku.edu.cn/), to analyze their respective gene structures in relation to intron-exon ratio. The gene structures were combined by the various Alba proteins distinctive motifs; the Alba protein motifs were determined by analyzing their respective protein sequences, through an online tool MEME, with default parameters set at 50 for maximum motif length and a minimum of 6, with the largest number of 15 (Brown et al., 2013). All the *Alba* genes were mapped into their respective chromosomes using the mapping tool, MapChart (Voorrips, 2002).

### Prediction of miRNA Targets and *Cis*-Regulatory Element Analysis in Cotton *Alba* Genes

In promoter sequences, 1,500 bp DNA sequence of each the *Alba* gene for the diploid cotton, *G. raimondii* was obtained from phytozome (www.phytozome.net), while for *G. hirsutum* then tetraploid cotton and that of *G. arboreum*, the diploid cotton of the A genome was obtained from the cotton functional database (https://cottonfgd.org). The *Alba* genes *cis*-regulatory elements were predicted by use of an online tool, the PLACE database (http://www.dna.affrc.go.jp/PLACE/signalscan.html), while the *Alba* genes targeted by miRNAs were predicted by using the online tool, the psRNATarget server with default parameters (http://plantgrn.noble.org/psRNATarget/).

### Plant Materials and Abiotic Stress Exposure

The seeds of the three cotton species, *G. hirsutum* (AD_1_), *G. raimondii* (D_5_), and *G. arboreum* (A_2_), were used. The three cotton germplasms are regularly maintained by our Institute of Cotton Research, Chinese Academy of Agricultural Sciences, CRI-CAAS. *G. hirsutum*, coded as CRI, was developed by our research institute and currently is the most preferred upland cotton grown in China; it covers more than 90% of the cotton growing regions. The seeds were delinted and then pre-treated before being germinated. Upon germination for 3 days, the seedlings were then transferred to a Hoagland solution (Hoagland and Arnon, 1950) in a hydroponic set up in the greenhouse, with temperature set at 28°C day/25°C night, 14 h photoperiod, and 60 to 70% relative humidity, a condition suitable for cotton growth. At three-leaf stages, the seedlings were exposed to water deficit and salinity stress, in which water deficit was initiated by transferring the seedlings into Hoagland nutrient solution supplemented with 15% of PEG-600 and samples collected at 0, 3, 6, 12, and 24 h, while salinity stress was imposed by supplementing the Hoagland solution with 250 mM of NaCl and samples collected at 0, 3, 6, 12, and 24 h. The tissues collected for both RNA extraction and expression analysis were root, leaf, and stem tissues.

### RNA Isolation and Real-Time Quantitative Polymerase Chain Reaction (RT-qPCR) Analysis

Total RNA was extracted from the two organs: leaf and root tissues of both treated and control plants under the two forms of abiotic stress condition, water deficit, and salt stress by using TRI reagent (Sigma Life Science, St. Louis, MO, USA). The quality and quantity of the RNA samples extracted were evaluated through NanoDrop 1000 (Thermo Fisher Scientific, Wilmington, DE, USA) and agarose gel electrophoresis. The cDNAs were synthesized using RNA samples with quality and quantity value of 260/280 ratio between 1.8 and 2.1, and 260/230 ratio between 2.0 and 2.5; all the RNA samples which felt out of the range were discarded and not used due to protein contamination. The *Alba* gene-specific primers were designed by Primer Premier 5 with melting temperatures of 55–60°C, primer lengths of 18–25 bp, and amplicon lengths of 101–221 bp (**Table S2**). Three biological replicates from each of the treatment, under drought, and salt stress, which comprised of three technical replicates, were analyzed. The transcript analysis was conducted by RT-qPCR using an ABI Prism 7500 Detection System (Applied Biosystems, Foster City, CA, USA). The cotton *GhActin* gene was used as the internal reference gene. The RT-qPCR reaction mixtures were carried out in a volume of 20 μl, containing 10 μl of SYBR Green Master Mix (Takara, Beijing, China), 2 μl of cDNA template, 6 μl of ddH_2_O, and 2 μl of each primer to make a final concentration of 10 μM. Reaction conditions were carried out with 95°C for 10 min, followed by 40 cycles of 95°C for 5 s, 59°C for 15 s, and 72°C for 30 s. The relative gene expression levels were calculated using the 2^− ΔΔCt^ methods (Rao et al., 2013).

### Generation of Transiently Transformed *G. hirsutum* Plants With Repression of *Gh_A01G0884* (ghAlba_4) and *Gh_D01G0922* (ghAlba_5) Genes

In this study, two types of transiently transformed *G. hirsutum* plants were generated in order to verify the function of *Alba* genes. For the VIG silencing of *Gh_A01G0884 (ghAlba_4)* and *Gh_D01G0922 (ghAlba_5)*, the tobacco rattle virus based VIGS technique was employed (Mu et al., 2016). The CDS of the two genes where used in designing the primers, 402 and 373 bp gene-specific fragments from *Gh_A01G0884 (ghAlba_4)* and *Gh_D01G0922 (ghAlba_5)*, respectively, were amplified by PCR using the v*Gh_A01G0884* and v*Gh_D01G0922* gene-specific primers (**Table S2**). The PCR products were cloned into the pTRV2 vector to produce TRV: *Gh_A01G0884 (ghAlba_4)* and TRV: *Gh_D01G0922 (ghAlba_5)* constructs. The *Agrobacterium tumefaciens*, strain LBA4404, was used in transforming the recombinant plasmids pTRV1, pTRV2, and the two TRV: *Alba* constructs, the process followed as outlined by Gao et al. (Gao et al., 2011b). After 2 weeks, samples were collected from the wild types, the positive control, and the VIGS plants. At three-leaf stage, water deficit and salt stress treatments were initiated. For water deficit conditions, irrigation was totally withdrawn, then after 6 days, various morphological, physiological, and biochemical parameters were determined. Moreover, for salt stress, the VIGS plant seedlings were irrigated with water supplemented with 250 mM of NaCl solution for a period of 4 days; thereafter, evaluation was carried out.

### Evaluation of Physiological Traits in the Two Transformed *G. hirsutum* Seedlings Under Stress Conditions

The cell membrane stability (CMS), excised leaf water loss (ELWL), and relative leaf water content (RLWC) were evaluated. The three physiological traits evaluated have been explored widely in determining the tolerance levels of plants to various abiotic stress—for instance, RLWC helps in evaluating plant water status (Tanentzap et al., 2015) and water stress and increased high levels of electrolytes affected the integrity of the cell membrane (Petrov et al., 2018). The CMS was determined as outlined by Fokar (Fokar et al., 1998). The RLWC determination was carried out as outlined by Barrs (Barrs and Weatherley, 1962) and, lastly, ELWL (McCaig and Romagosa, 1989). All the measurements were carried out in three biological repeats.

### Evaluation of Chlorophyll Content, Oxidants, and Antioxidant in the Transformed and Non-Transformed *G. hirsutum* Cotton Plant Under Stress Conditions

In order to determine the role of the *Alba* genes in cotton, we examined the chlorophyll content, oxidants, and antioxidant levels within the transformed seedlings and the non-transformed ones under salt, and water deficit conditions. Just as described in section 2.8, after 24 h of stress exposure, we measured the leaf chlorophyll content using MINOLTA SPAD, a non-destructive method (do et al., 2008). The oxidants and antioxidants were determined as described by Lu et al. (Lu et al., 2018b), in their analysis of *GPCR* genes role under salt stress condition in transgenic *Arabidopsis* plants. We evaluated three antioxidants, SOD, CAT, and POD. The CAT antioxidant enzyme activity was evaluated by determining the reducing level of H_2_O_2_, as outlined by Cakmak and Marschner (Cakmak and Marschner, 1992); SOD activity was evaluated by the determination of the inhibition of the photochemical reduction of nitro blue tetrazoliumas described by Giannopolitis and Ries (Giannopolitis and Ries, 1977), and finally the POD evaluated as described by Van Assche (Van Assche et al., 1988). The MDA and H_2_O_2_ were evaluated between the two plants, non-transformed, and the transformed under the three stress levels. MDA was determined a measure of lipid peroxidation (Cakmak and Horst, 1991), and H_2_O_2_ concentration was measured as described by Loreto and Velikova (Loreto and Velikova, 2001).

### Root Assays of the Two Cotton Plants, the VIGS, and Wild Type (WT) Under Water Deficit and Salt Stress Conditions

In order to evaluate the effect of knockdown of the two *Alba* genes on the plant root growth, vermiculite and sand were used as the rooting medium. The roots were evaluated after 14 days of stress exposure, the roots were carefully removed from the rooting medium and washed, and various parameters were measured. The root evaluation was carried out by determining the root length, by the use of root scanner, WinRHIZO Epson V700 Photo Scanner JZZIA (model number expression 11000XL), obtained from Seiko Epson Corp. Japan. The dry weights were determined after oven drying at 80°C.

### Statistical Analysis

All the experimental data derived from this research were computed from the mean values of three biological replicates, and statistical analysis was carried out using IBM SPSS Statistics 20. The variations between the VIGS cotton and the control plants under water deficit and salt stress treatment were evaluated by one way ANOVA.

## Results

### Identification of the Alba Proteins in Cotton Species

A total of 60 proteins encoded by the cotton *Alba* genes were determined and found to be distributed across the three cotton genomes, with 33, 20, and 17 Alba proteins in *G. hirsutum* (AD_1_), *G. raimondii* (D_5_), and *G. arboreum* (A_2_), respectively. The Alba proteins in the three cotton species were confirmed both through SMART and Pfam scan for all the sequences obtained from the HMM profile and BLASTP search. The number of Alba proteins in *G. hirsutum* was higher than in either of the two diploid cotton species, though an element of gene loss was detected, being *G. hirsutum* evolved through whole genome duplication (WGD) between A and D genomes (Li et al., 2016). The cotton Alba protein lengths ranged from 62 to 312 aa, in which the highest protein lengths were detected in *G. hirsutum* and *G. raimondii* with both having the highest protein lengths of 312 aa, for *Gh_A04G1077* (*RPP25L*) and *Gorai.012G160400* (*RPP25L*) in *G. hirsutum* and *G. raimondii*, respectively. The two Alba proteins were all members of ribonuclease P protein subunit p25-like protein (*RPP25L*), being in agreement with the previous results in rice with the highest and lowest protein lengths of 320 and 132 aa, respectively (Kumar Verma et al., 2018).

The other physiochemical properties for the cotton Alba proteins were varied across the three cotton species. In *G. hirsutum*, the molecular weight (MW) ranged from 7.003 to 34.55 kDa, the electric charge from −3 to 21.5, isoelectric (*pI*) values from 4.761 to 10.91 and the grand average hydropathy (GRAVY) values from −1.012 to 0.311. In *G. arboreum*, their protein lengths ranged from 122 to 284 aa, MW from 13 to 32 kDa, a charge of −3 to 19, *pI* values from 5 to 11, and the GRAVY values of −0.972 to −0.183. Finally, in *G. raimondii*, Alba protein lengths ranged from 90 to 312 aa, MW from 10 to 34 kDa, *pI* values from 5 to 11, and the GRAVY values of −0.985 to 0.609 (**Table 1**). Over 99% of the cotton Alba proteins had GRAVY values below zero, which showed that they are hydrophilic, a property shared among the proteins encoded by the various stress-responsive genes such as the *LEA* (Magwanga et al., 2018), *GPCR* (Lu et al., 2018b), and *MATE* (Lu et al., 2018a).

**Table 1:** Identification, gene annotation, and physiochemical properties of the cotton *Alba* gene.

Genome	Gene ID	Alba gene annotation	Clade group	Length (aa)	MW (kDa)	Charge	pI	GRAVY
*G. arboreum* (AA)	Cotton_A_02961	*cottAlba_9*	Clade_2	218	24.259	19	10.7	−0.911
	Cotton_A_03076	*cottAlba_15*	Clade_4	132	14.771	−2.5	5.012	−0.39
	Cotton_A_03232	*cottAlba_8*	Clade_2	260	28.653	18.5	10.36	−0.866
	Cotton_A_08372	*cottAlba_10*	Clade_3	190	20.76	1	8.182	−0.319
	Cotton_A_08836	*cottAlba_6*	Clade_1	241	25.991	14.5	10.73	−0.909
	Cotton_A_10898	*cottAlba_4*	Clade_1	253	27.6	16	10.59	−0.866
	Cotton_A_11444	*cottAlba_12*	Clade_4	125	13.905	1	7.688	−0.333
	Cotton_A_14221	*cottAlba_2*	Clade_1	197	22.023	15.5	10.47	−0.972
	Cotton_A_16250	*cottAlba_16*	Clade_4	122	13.402	−3	4.761	−0.27
	Cotton_A_16451	*cottAlba_1*	Clade_1	248	27.045	15	10.64	−0.822
	Cotton_A_17439	*cottAlba_13*	Clade_4	154	17.29	3	8.992	−0.551
	Cotton_A_20171	*cottAlba_14*	Clade_4	134	14.854	−3	4.8	−0.396
	Cotton_A_28567	*cottAlba_3*	Clade_1	251	27.864	17	10.85	−0.953
	Cotton_A_30556	*cottAlba_11*	Clade_4	130	14.002	4.5	9.992	−0.183
	Cotton_A_33889	*cottAlba_7*	Clade_1	257	27.527	13.5	10.5	−0.925
	Cotton_A_36717	*cottAlba_17*	Clade_4	131	14.41	−1	5.292	−0.308
	Cotton_A_40133	*cottAlba_5*	Clade_1	284	31.586	8	9.589	−0.329
*G. hirsutum* (AADD)	Gh_A01G0884	*ghAlba_4*	Clade_1	251	27.864	17	10.85	−0.953
	Gh_A01G1470	*ghAlba_13*	Clade_1	257	27.533	12.5	10.41	−0.925
	Gh_A02G0345	*ghAlba_20*	Clade_3	183	20.04	1	8.434	−0.374
	Gh_A04G1077	*ghAlba_14*	Clade_1	312	34.55	12.5	9.784	−0.406
	Gh_A05G0101	*ghAlba_27*	Clade_4	130	14.498	0	6.545	−0.391
	Gh_A05G1575	*ghAlba_1*	Clade_1	250	27.289	16	10.74	−0.836
	Gh_A05G3960	*ghalba_31*	Clade_4	122	13.402	−3	4.761	−0.27
	Gh_A06G0483	*ghAlba_26*	Clade_4	130	14.488	−1	5.806	−0.355
	Gh_A08G0430	*ghAlba_16*	Clade_2	238	26.268	19.5	10.63	−1.012
	Gh_A08G2091	*ghAlba_32*	Clade_4	134	14.826	−3	4.8	−0.413
	Gh_A11G0507	*ghAlba_6*	Clade_1	249	27.186	16	10.59	−0.814
	Gh_A11G1257	*ghAlba_18*	Clade_2	239	26.624	21.5	10.72	−0.977
	Gh_A11G2262	*ghAlba_24*	Clade_4	130	14.003	2.5	9.497	−0.18
	Gh_A12G0762	*ghAlba_9*	Clade_1	272	29.547	7	9.751	−0.516
	Gh_A13G1770	*ghAlba_29*	Clade_4	132	14.771	−2.5	5.012	−0.39
	Gh_D01G0359	*ghAlba_3*	Clade_1	228	25.23	15.5	10.62	−0.905
	Gh_D01G0922	*ghAlba_5*	Clade_1	251	27.833	16	10.89	−0.904
	Gh_D01G1707	*ghAlba_11*	Clade_1	254	27.064	13	10.32	−0.866
	Gh_D02G0408	*ghAlba_21*	Clade_3	183	20.039	3	9.41	−0.356
	Gh_D03G0593	*ghAlba_22*	Clade_4	132	14.436	1	8.895	−0.251
	Gh_D03G1718	*ghAlba_10*	Clade_1	62	7.003	2	9.263	0.311
	Gh_D04G2019	*ghAlba_12*	Clade_1	312	34.523	15.5	10.09	−0.44
	Gh_D05G0083	*ghAlba_28*	Clade_4	122	13.429	−3	4.761	−0.292
	Gh_D05G1753	ghAlba_2	Clade 1	251	27.367	18.5	10.91	−0.781
	Gh_D06G0537	ghAlba_25	Clade_4	130	14.488	−1	5.806	−0.36
	Gh_D08G0518	ghAlba_17	Clade_2	213	23.651	13.5	10.27	−0.9
	Gh_D08G2460	ghAlba_33	Clade_4	134	14.814	−3	4.8	−0.387
	Gh_D11G0585	ghAlba_7	Clade_1	249	27.229	17	10.67	−0.825
	Gh_D11G1406	ghAlba_19	Clade_2	239	26.568	21.5	10.72	−0.958
	Gh_D11G2569	ghAlba_23	Clade_4	132	14.116	4.5	9.992	−0.186
	Gh_D12G0886	ghAlba_8	Clade_1	252	27.501	11	10.2	−0.679
	Gh_D13G2120	ghalba_30	Clade_4	132	14.771	−2.5	5.012	−0.39
	Gh_Sca129121G01	ghAlba_15	Clade_1	68	7.763	4	10.18	−0.478
G. raimondii (DD)	Gorai.002G047400	Gorai_2	Clade_1	228	25.204	15.5	10.62	−0.925
	Gorai.002G121600	Gorai_3	Clade_1	252	27.902	16	10.89	−0.914
	Gorai.002G140000	Gorai_6	Clade_1	118	13.366	4	9.764	0.131
	Gorai.002G206900	Gorai_10	Clade_1	259	27.761	14	10.5	−0.926
	Gorai.003G023400	Gorai_8	Clde_1	118	13.338	3	9.488	0.181
	Gorai.004G058400	Gorai_11	Clade_2	238	26.421	19	10.43	−0.985
	Gorai.004G274000	Gorai_18	Clade_4	134	14.854	−3	4.8	−0.35
	Gorai.005G046900	Gorai_13	Clade_3	183	20.012	2	9.017	−0.339
	Gorai.007G063100	Gorai_4	Clade_1	249	27.183	17	10.67	−0.837
	Gorai.007G153000	Gorai_12	Clade_2	239	26.538	22.5	10.8	−0.973
	Gorai.007G278700	Gorai_14	Clade_4	138	14.459	4.5	9.992	−0.196
	Gorai.008G100300	Gorai_5	Clade_1	254	27.737	13	10.48	−0.744
	Gorai.009G010100	Gorai_17	Clade_4	122	13.413	−2	4.945	−0.229
	Gorai.009G018500	Gorai_16	Clade_4	130	14.496	−0.5	6.268	−0.315
	Gorai.009G192000	Gorai_1	Clade _1	251	27.419	17.5	10.81	−0.797
	Gorai.010G064500	Gorai_15	Clade_4	130	14.464	−1.5	5.293	−0.301
	Gorai.012G160400	Gorai_9	Clade_1	312	34.479	15.5	10.09	−0.432
	Gorai.013G105600	Gorai_19	Clade_4	132	14.495	1	8.885	−0.257
	Gorai.013G106200	Gorai_7	Clade_1	90	10.04	4	9.34	0.609
	Gorai.013G233800	Gorai_20	Clade_4	132	14.787	−2.5	5.012	−0.41

aa, amino acid; MW, molecular weight in KiloDalton; pI, isoelectric point; GRAVY, grand average hydropathy values. The enclosed genes were further used in gene induced silencing for functional characterization under drought and salt stress conditions.

### Phylogenetic Tree Analysis, Chromosomal Mapping, and Subcellular Localization Prediction of the Cotton Alba Proteins

By integrating all the three cotton Alba protein sequences together with *O. sativa*, *T. cacao*, *A. thaliana*, *S. bicolor*, *Populus trichocarpa*, and *G. max* were aligned them through Clustal and constructed phylogenetic tree by MEGA 7. The Alba proteins from cotton and the other plants were clustered into four distinct groups, designated as clade 1 to clade 4. Clade 4 was the largest, then closely followed by clade1, while clades 2 and 3 had a fewer number of Alba proteins (**Figure S1**). In all the clades, cotton proteins encoded by the *Alba* genes were found to have an orthologous gene pair with other plants, though the majority of the ortholog genes were formed between the three proteins encoded by *Alba* genes derived from the three cotton species. In clade 1, the orthologous gene pairs between the cotton *Alba* genes were *Gorai.002G121600* and *AT1G76010* and *Glyma.06G148800* and *Gh_A12G0762*, and the third orthologous pair were *Thecc1EG006429* and *Gorai.012G160400*.

In clades 2 and 3, no ortholog gene pairs were formed between the cotton *Alba* genes with any other plant used in the phylogenetic tree analysis. In clade 4, *Thecc1EG038396* and *Gh_D06G0537*, *Cotton_A_30556*, and *Thecc1EG026310* were the only ortholog gene pairs between the cotton and the other plants; the rest of the ortholog gene pairs were between the cotton proteins encoded by *Alba* genes. The detection of the ortholog gene pairs between the cotton proteins encoded by the *Alba* genes showed that these proteins might have evolved from a common origin. *T. cacao* and *Gossypium* species shared a common evolutionary origin. The results obtained in the analysis of the phylogeny of the cotton *Alba* genes are in agreement with previous reports in rice with similar domain compositions clustered in the same clade (Kumar Verma et al., 2018).

The distribution of the *Alba* genes across the 26 chromosomes of the tetraploid cotton was uneven, only 19 chromosomes out of the 26 chromosomes in tetraploid cotton; *G. hirsutum* were found to harbor the *Alba* genes. The highest loci density among the mapped chromosomes in the tetraploid cotton was noted in chromosomes A_h_05, A_h_11, D_h_01, and D_h_11 with three genes each. The following chromosomes harbored no *Alba* genes: chrA_h_03, chrA_h_07, chrA_h_09, chrA_h_10, chrD_h_07, chrD_h_09, and chrD_h_10. The most interesting is that three sets of chromosomes, chrA_h_07, chrA_h_09, and chrA_h_10 and their corresponding homologs harbored no genes. The uneven distribution of the *Alba* genes could be attributed to their low numbers, only 33 genes in a relatively large genome of the tetraploid cotton, *G. hirsutum*. In the diploid cotton, 10 and 11 chromosomes were found to harbor the *Alba* genes in *G. raimondii* and *G. arboreum*, respectively. In *G. raimondii*, the highest gene loci were observed in chrD_5_02, with four genes; chrD_5_07, chrD_5_09, and chrD_5_10, with three genes each; and chrD_5_03, chrD_5_05, chrD_5_08, chrD_5_10, and chrD_5_12, with a single gene each. No genes were detected to be mapped in chrD_5_01, chrD_5_06, and chrD_5_11. In the diploid cotton of the A genome, the highest gene loci were detected in chrA_2_02, chrA_2_03, chrA_2_06, chr A_2_08, and chrA_2_12 with two genes in each; the rest of the mapped chromosomes, chr A_2_04, chrA_2_05, chrA_2_09, chrA_2_10, and chrA_2_13, had a gene in each. The chromosomes were named as described by Wang et al. (Kunbo et al., 2018).

In the analysis of subcellular predictions, a higher percentage of the proteins encoded by the *Alba* genes was found to be located within the nucleus, which was evident across the three cotton species. Among the proteins encoded by the *Alba* genes, obtained for the tetraploid cotton, out of the 33 proteins, 16 were found to be located within the nucleus, 6 endoplasmic reticulum (E.R), 4 cytoplasm, 3 in the extracellular structures (Extr), 3 within the mitochondrion, and 1 within the plasma membrane. In the diploid cotton of D genome, *G. raimondii*, 13 proteins encoded by the *Alba* genes were predicted to be located within the nucleus, 3 in E.R, and 2 in the cytoplasm, 1 in extracellular structures, and 1 in the mitochondrion. Finally, in *G. arboreum*, nine proteins encoded by the *Alba* genes were predicted to be localized within the nucleus; four in the E.R, two in the cytoplasm, and one each were found to be embedded in the extracellular structures and mitochondrion (**Table 2**). The subcellular localization predictions of the cotton Alba proteins encoded by the *Alba* genes showed that the majority of the cotton alba proteins are located within the nucleus, 48.5, 65, and 52% of all the Alba proteins encoded by the *Alba* genes in *G. hirsutum*, *G. raimondii*, and *G. arboreum*, respectively. The high number of the proteins encoded by the *alba* genes embedded within the nucleus could possibly mean that these proteins could be playing an integral role within the nucleus, in relation to gene expression regulation, more so stress-responsive genes (Verma et al., 2014).

**Table 2:** Identification and subcellular localization prediction of the cotton Alba proteins.

Genome	Gene ID	Gene annotation	Clade number	Gene name	Description	Chr.	Start	End	Length (bp)	WolF SPORT
**AADD**	Gh_A01G0884	*ghAlba_4*	Clade_1	*Rpp25l*	Ribonuclease P protein subunit p25-like protein	A01	20,840,531	20,842,428	1,898	nucl
	Gh_A01G1470	*ghAlba_13*	Clade_1	*RPP25L*	Ribonuclease P protein subunit p25-like protein	A01	89,998,128	90,000,798	2,671	nucl
	Gh_A02G0345	*ghAlba_20*	Clade_3	*At2g34160*	Uncharacterized protein At2g34160	A02	4,060,146	4,061,559	1,414	cyto
	Gh_A04G1077	*ghAlba_14*	Clade_1	*RPP25L*	Ribonuclease P protein subunit p25-like protein	A04	60,858,552	60,861,349	2,798	nucl
	Gh_A05G0101	*ghAlba_27*	Clade_4	*At2g34160*	Uncharacterized protein At2g34160	A05	1,200,258	1,201,591	1,334	nucl
	Gh_A05G1575	*ghAlba_1*	Clade_1	*Rpp25l*	Ribonuclease P protein subunit p25-like protein	A05	16,150,964	16,152,864	1,901	nucl
	Gh_A05G3960	*ghalba_31*	Clade_4	*At2g34160*	Uncharacterized protein At2g34160	A05	51,864	53,267	1,404	cyto
	Gh_A06G0483	*ghAlba_26*	Clade_4	*At2g34160*	Uncharacterized protein At2g34160	A06	9,399,140	9,400,592	1,453	nucl
	Gh_A08G0430	*ghAlba_16*	Clade_2	*RPP25L*	Ribonuclease P protein subunit p25-like protein	A08	5,667,356	5,669,691	2,336	E.R.
	Gh_A08G2091	*ghAlba_32*	Clade_4	*At2g34160*	Uncharacterized protein At2g34160	A08	102,166,731	102,168,710	1,980	E.R.
	Gh_A11G0507	*ghAlba_6*	Clade_1	*Rpp25l*	Ribonuclease P protein subunit p25-like protein	A11	4,756,389	4,758,020	1,632	nucl
	Gh_A11G1257	*ghAlba_18*	Clade_2	*RPP25L*	Ribonuclease P protein subunit p25-like protein	A11	15,650,000	15,652,543	2,544	E.R.
	Gh_A11G2262	*ghAlba_24*	Clade_4	*At2g34160*	Uncharacterized protein At2g34160	A11	77,668,711	77,669,861	1,151	mito
	Gh_A12G0762	*ghAlba_9*	Clade_1	*Rpp25l*	Ribonuclease P protein subunit p25-like protein	A12	40,181,865	40,185,742	3,878	nucl
	Gh_A13G1770	*ghAlba_29*	Clade_4	*At2g34160*	Uncharacterized protein At2g34160	A13	76,646,644	76,648,135	1,492	extr
	Gh_D01G0359	*ghAlba_3*	Clade_1	*Rpp25l*	Ribonuclease P protein subunit p25-like protein	D01	4,033,473	4,034,159	687	nucl
	Gh_D01G0922	*ghAlba_5*	Clade_1	*Rpp25l*	Ribonuclease P protein subunit p25-like protein	D01	15,410,641	15,412,496	1,856	nucl
	Gh_D01G1707	*ghAlba_11*	Clade_1	*RPP25L*	Ribonuclease P protein subunit p25-like protein	D01	53,649,790	53,652,471	2,682	nucl
	Gh_D02G0408	*ghAlba_21*	Clade_3	*At2g34160*	Uncharacterized protein At2g34160	D02	5,263,552	5,264,943	1,392	cyto
	Gh_D03G0593	*ghAlba_22*	Clade_4	*At2g34160*	Uncharacterized protein At2g34160	D03	13,552,172	13,554,154	1,983	plas
	Gh_D03G1718	*ghAlba_10*	Clade_1	*NA*	NA	D03	797,217	797,568	352	mito
	Gh_D04G2019	*ghAlba_12*	Clade_1	*RPP25L*	Ribonuclease P protein subunit p25-like protein	D04	34,081	36,874	2,794	nucl
	Gh_D05G0083	*ghAlba_28*	Clade_4	*At2g34160*	Uncharacterized protein At2g34160	D05	876,193	877,643	1,451	cyto
	Gh_D05G1753	*ghAlba_2*	Clade 1	*Rpp25l*	Ribonuclease P protein subunit p25-like protein	D05	15,825,693	15,827,603	1,911	nucl
	Gh_D06G0537	*ghAlba_25*	Clade_4	*At2g34160*	Uncharacterized protein At2g34160	D06	8,184,698	8,186,141	1,444	extr
	Gh_D08G0518	*ghAlba_17*	Clade_2	*RPP25L*	Ribonuclease P protein subunit p25-like protein	D08	5,822,524	5,824,866	2,343	E.R.
	Gh_D08G2460	*ghAlba_33*	Clade_4	*At2g34160*	Uncharacterized protein At2g34160	D08	64,516,539	64,518,502	1,964	E.R.
	Gh_D11G0585	*ghAlba_7*	Clade_1	*Rpp25l*	Ribonuclease P protein subunit p25-like protein	D11	5,006,767	5,008,401	1,635	nucl
	Gh_D11G1406	*ghAlba_19*	Clade_2	*RPP25L*	Ribonuclease P protein subunit p25-like protein	D11	13,834,200	13,836,773	2,574	E.R.
	Gh_D11G2569	*ghAlba_23*	Clade_4	*At2g34160*	Uncharacterized protein At2g34160	D11	53,216,229	53,217,381	1,153	mito
	Gh_D12G0886	*ghAlba_8*	Clade_1	*Rpp25l*	Ribonuclease P protein subunit p25-like protein	D12	30,394,300	30,398,183	3,884	nucl
	Gh_D13G2120	*ghalba_30*	Clade_4	*At2g34160*	Uncharacterized protein At2g34160	D13	56,818,765	56,820,250	1,486	extr
	Gh_Sca129121G01	*ghAlba_15*	Clade_1	*NA*	NA	scaffold	63	355	293	nucl
**DD**	Gorai.002G047400	*Gorai_2*	Clade_1	*Rpp25l*	Ribonuclease P protein subunit p25-like protein	Chr02	4,006,829	4,007,515	687	nucl
	Gorai.002G121600	*Gorai_3*	Clade_1	*Rpp25l*	Ribonuclease P protein subunit p25-like protein	Chr02	17,446,507	17,449,331	2,825	nucl
	Gorai.002G140000	*Gorai_6*	Clade_1	*NA*	NA	Chr02	24,300,823	24,301,909	1,087	nucl
	Gorai.002G206900	*Gorai_10*	Clade_1	*RPP25L*	Ribonuclease P protein subunit p25-like protein	Chr02	55,304,459	55,307,818	3,360	nucl
	Gorai.003G023400	*Gorai_8*	Clde_1	*NA*	NA	Chr03	1,807,535	1,811,458	3,924	nucl
	Gorai.004G058400	*Gorai_11*	Clade_2	*RPP25L*	Ribonuclease P protein subunit p25-like protein	Chr04	5,640,843	5,643,721	2,879	nucl
	Gorai.004G274000	*Gorai_18*	Clade_4	*At2g34160*	Uncharacterized protein At2g34160	Chr04	60,853,194	60,855,717	2,524	E.R.
	Gorai.005G046900	*Gorai_13*	Clade_3	*At2g34160*	Uncharacterized protein At2g34160	Chr05	4,441,843	4,444,342	2,500	cyto
	Gorai.007G063100	*Gorai_4*	Clade_1	*Rpp25l*	Ribonuclease P protein subunit p25-like protein	Chr07	4,426,542	4,428,637	2,096	nucl
	Gorai.007G153000	*Gorai_12*	Clade_2	*RPP25L*	Ribonuclease P protein subunit p25-like protein	Chr07	13,081,429	13,084,954	3,526	E.R.
	Gorai.007G278700	*Gorai_14*	Clade_4	*At2g34160*	Uncharacterized protein At2g34160	Chr07	47,671,674	47,673,365	1,692	mito
	Gorai.008G100300	*Gorai_5*	Clade_1	*Rpp25l*	Ribonuclease P protein subunit p25-like protein	Chr08	29,524,423	29,529,261	4,839	nucl
	Gorai.009G010100	*Gorai_17*	Clade_4	*At2g34160*	Uncharacterized protein At2g34160	Chr09	812,133	813,985	1,853	cyto
	Gorai.009G018500	*Gorai_16*	Clade_4	*At2g34160*	Uncharacterized protein At2g34160	Chr09	1,445,511	1,447,488	1,978	nucl
	Gorai.009G192000	*Gorai_1*	Clade _1	*Rpp25l*	Ribonuclease P protein subunit p25-like protein	Chr09	14,777,906	14,780,753	2,848	nucl
	Gorai.010G064500	*Gorai_15*	Clade_4	*At2g34160*	Uncharacterized protein At2g34160	Chr10	8,209,481	8,211,614	2,134	extr
	Gorai.012G160400	*Gorai_9*	Clade_1	*RPP25L*	Ribonuclease P protein subunit p25-like protein	Chr12	33,295,789	33,299,056	3,268	nucl
	Gorai.013G105600	*Gorai_19*	Clade_4	*At2g34160*	Uncharacterized protein At2g34160	Chr13	21,452,061	21,454,549	2,489	E.R.
	Gorai.013G106200	*Gorai_7*	Clade_1	*NA*	NA	Chr13	22,565,006	22,566,245	1,240	nucl
	Gorai.013G233800	*Gorai_20*	Clade_4	*At2g34160*	Uncharacterized protein At2g34160	Chr13	55,231,328	55,233,248	1,921	nucl
**AA**	Cotton_A_02961	*cottAlba_9*	Clade_2	*RPP25L*	Ribonuclease P protein subunit p25-like protein	Chr11	108,092,269	108,094,812	2,544	E.R.
	Cotton_A_03076	*cottAlba_15*	Clade_4	*At2g34160*	Uncharacterized protein At2g34160	Chr13	120,084,369	120,085,861	1,493	extr
	Cotton_A_03232	*cottAlba_8*	Clade_2	*RPP25L*	Ribonuclease P protein subunit p25-like protein	Chr08	6,900,181	6,902,537	2,357	E.R.
	Cotton_A_08372	*cottAlba_10*	Clade_3	*At2g34160*	Uncharacterized protein At2g34160	Chr03	4,515,978	4,517,404	1,427	cyto
	Cotton_A_08836	*cottAlba_6*	Clade_1	*RPP25L*	Ribonuclease P protein subunit p25-like protein	Chr04	2,678,336	2,681,021	2,686	nucl
	Cotton_A_10898	*cottAlba_4*	Clade_1	*Rpp25l*	Ribonuclease P protein subunit p25-like protein	Chr11	119,273,112	119,274,743	1,632	E.R.
	Cotton_A_11444	*cottAlba_12*	Clade_4	*At2g34160*	Uncharacterized protein At2g34160	Chr05	1,643,818	1,645,132	1,315	nucl
	Cotton_A_14221	*cottAlba_2*	Clade_1	*Rpp25l*	Ribonuclease P protein subunit p25-like protein	Chr01	5,248,520	5,249,201	682	nucl
	Cotton_A_16250	*cottAlba_16*	Clade_4	*At2g34160*	Uncharacterized protein At2g34160	Chr05	974,452	975,859	1,408	cyto
	Cotton_A_16451	*cottAlba_1*	Clade_1	*Rpp25l*	Ribonuclease P protein subunit p25-like protein	Chr05	17,646,823	17,648,719	1,897	nucl
	Cotton_A_17439	*cottAlba_13*	Clade_4	*At2g34160*	Uncharacterized protein At2g34160	Chr06	9,063,402	9,064,857	1,456	nucl
	Cotton_A_20171	*cottAlba_14*	Clade_4	*At2g34160*	Uncharacterized protein At2g34160	Chr08	127,938,045	127,940,022	1,978	E.R.
	Cotton_A_28567	*cottAlba_3*	Clade_1	*Rpp25l*	Ribonuclease P protein subunit p25-like protein	Chr01	22,489,014	22,490,910	1,897	nucl
	Cotton_A_30556	*cottAlba_11*	Clade_4	*At2g34160*	Uncharacterized protein At2g34160	Chr11	19,319,670	19,320,822	1,153	mito
	Cotton_A_33889	*cottAlba_7*	Clade_1	*RPP25L*	Ribonuclease P protein subunit p25-like protein	Chr02	88,720,747	88,723,418	2,672	nucl
	Cotton_A_36717	*cottAlba_17*	Clade_4	*At2g34160*	Uncharacterized protein At2g34160	Chr02	78,452,904	78,454,608	1,705	nucl
	Cotton_A_40133	*cottAlba_5*	Clade_1	*Rpp25l*	Ribonuclease P protein subunit p25-like protein	Chr12	44,110,262	44,114,140	3,879	nucl

*Chr, chromosome; bp, base pair; nucl, nucleus; extr, extracellular structure; mito, mitochondrion; E.R, endoplasmic reticulum; cyto, cytoplas.*

### Gene Structure and Motif Identification

For the analysis of the *Alba* gene structures in the three cotton species, all the *Alba* genes were found to be disrupted, except two genes, *Gh_D01G0359* in the tetraploid cotton and *Gorai.002G047400* in diploid cotton of the D genome, which were intronless and members of the ribonuclease P protein subunit p25-like protein (*Rpp25l*). Among the *Alba* genes in the tetraploid cotton, the lowest intron disruption of only one was observed in two genes, *Gh_D03G1718* and *Gh_Sca129121G01*, while the highest intron disruption of eight was detected in *Gh_A12G0762* (**Figure S2A**). In *G. raimondii*, the highest level of intron disruption was detected in *Gorai.003G023400* with nine disruptions, and the least intron disruption was observed for *Gorai.002G140000*, with two introns (**Figure S2B**). Similarly, in *G. arboreum*, the least intron disruption was one, while the highest intron disruption was eight also, as observed in *Cotton_A_14221* and *Cotton_A_03232*, respectively (**Figure S2C**). These results were in agreement with the previous reports in rice, *sAlba1*, which was found to be interrupted by four introns (Verma et al., 2014). Cotton *Alba* genes had distinctive motifs. In the tetraploid cotton among 33 *Alba* genes, the following motifs were common: motif 1, motif 2, and motif 6. In *G. arboreum*, motif 1 and motif 2 were common among its all *Alba* genes, while in *G. raimondii*, motif 1 motif 2 and motif 5 were common among its all *Alba* genes. In combined analysis of all the three cotton *Alba* genes, very specific distinctive motifs were identified, which can be used for the identification and characterization of the *Alba* genes in cotton. The common motifs identified were motif 1 motif 2 and motif 3.

### miRNA Target and *Cis*-Regulatory Element Analysis of the Cotton *Alba* Genes

The plants small/micro ribonucleic acids (miRNAs) have emerged as a significant player in translational, transcriptional, and post-transcriptional regulation of plant genes, which are vital plant responsiveness to various kinds of abiotic and biotic stress factors (Kumar, 2014). In the analysis of the possible miRNAs targets to various cotton *Alba* genes, no miRNAs were detected to target any of the *Alba* genes obtained from *G. arboreum*; however, in the *G. raimondii*, a diploid cotton of the D genome, high level of miRNAs target, was observed; 52 miRNAs were found to target all the 20 *Alba* genes in *G. raimondii*. The miRNAs with the highest gene targets were gra-miR8770 that targeted 4 genes, gra-miR8752 and gra-miR8666 that targeted 3 genes each, and gra-miR8657a, b, c, d, and e that targeted 10 genes, while the rest targeted either 1 to a maximum of 2 genes each. Some of the *Alba* genes were found to be targeted by more than 5 miRNAs—for instance, *Gorai.002G206900* was targeted by 8 miRNAs, *Gorai.004G274000* was targeted by 6 miRNAs, *Gorai.007G063100* and *Gorai.012G160400* were targeted by 11 miRNAs, and *Gorai.013G105600* was targeted by 7 miRNAs. Low level of miRNAs targets was observed among the *Alba* genes obtained for the tetraploid cotton, *G. hirsutum*; only 16 miRNAs were found to target 17 genes; the genes with the highest miRNA target were *Gh_A04G1077*, *Gh_A05G1575*, *Gh_A11G2262*, *Gh_D04G2019*, and *Gh_D05G1753* with three miRNA each; and the rest of the genes were either target by 1 to 2 miRNAs. Only one miRNA, ghr-miR7498, was found to target four genes, such as *Gh_A05G3960*, *Gh_A11G2262*, *Gh_D05G0083*, and *Gh_D11G2569* (**Table S3**). One of the most significant miRNA detected was ghr-miR394a; the same miRNA has been found to be highly upregulated in *Arabidopsis* under water deficit condition (Liu et al., 2009).

Plant responses and acclimations under various environmental stress factors require differential gene expression, which is modulated by a given plant transcription factor (TF) (Saibo et al., 2008; Banerjee and Roychoudhury, 2017; Hernandez and Sanan-Mishra, 2017). The TFs are proteins with a DNA domain that binds to the *cis*-regulatory element found within the promoter regions of the targeted gene (Song et al., 2001). Several plant TFs have been identified—for instance, abscisic acid (ABA) responsive element (ABRE), CBF/DREB, myeloblastosis (MYB/MYC), AP2/ERF, and the NAM, ATAF1/2, and CUC2 (NAC) domain, which are the major plant-specific families of the TFs with significant role in the regulation of the abiotic stress-induced multiple gene expression in an ABA-dependent or independent processes (Qin et al., 2011). The most abundant forms of the *cis*-regulatory elements detected across the three cotton species were CAATBOX1 (disease resistance/putative functions in response to environmental stresses), GATABOX (required for high level, light-regulated, and tissue-specific expression), MYCCONSENSUSAT (MYC recognition site found in the promoters of the dehydration-responsive gene rd22), GT1CONSENSUS (light regulation), WRKY71OS (positive and negative regulators of ABA signaling), MYBCORE (dehydration/ water stress), and ABRELATERD1 (function in induction by dehydration stress and dark-induced senescence) (**Figure S3** and **Table S4**). The detection of these *cis*-regulatory elements showed that the cotton *Alba* genes indicated their broader functions in enhancing abiotic and biotic stress tolerances.

### RNA Sequence Data Analysis and RT-qPCR Validation of the Various *Alba* Genes Under Salt and Water Deficit Conditions

The RNA sequence profiling of leaf, root, and stem under water deficit and salt stress conditions showed that the upland cotton, *G. hirsutum Alba* genes, were grouped into three groups as per their expression pattern. In both salt and water deficit conditions, group 1 gene exhibited significant upregulations across all the tissues tested and in different time points of stress exposure. Group 2 showed differential expression, though in most of the time points, more were up-regulated. In group 3, they were either down-regulated or showed no expressions at all in all the tissues tested. Some of the genes were found to have significant upregulation under salt and water deficit, such as *Gh_D05G0083* (DNA-/RNA-binding protein), *Gh_A08G2091* (DNA-/RNA-binding protein), *Gh_D01G0922* (ribonuclease P protein), *Gh_A01G0884* (ribonuclease P protein), *Gh_D13G2120* (DNA-/RNA-binding protein), and *Gh_A13G1770* (DNA-/RNA-binding protein) (**Figure S4**).

**Figure 1:**
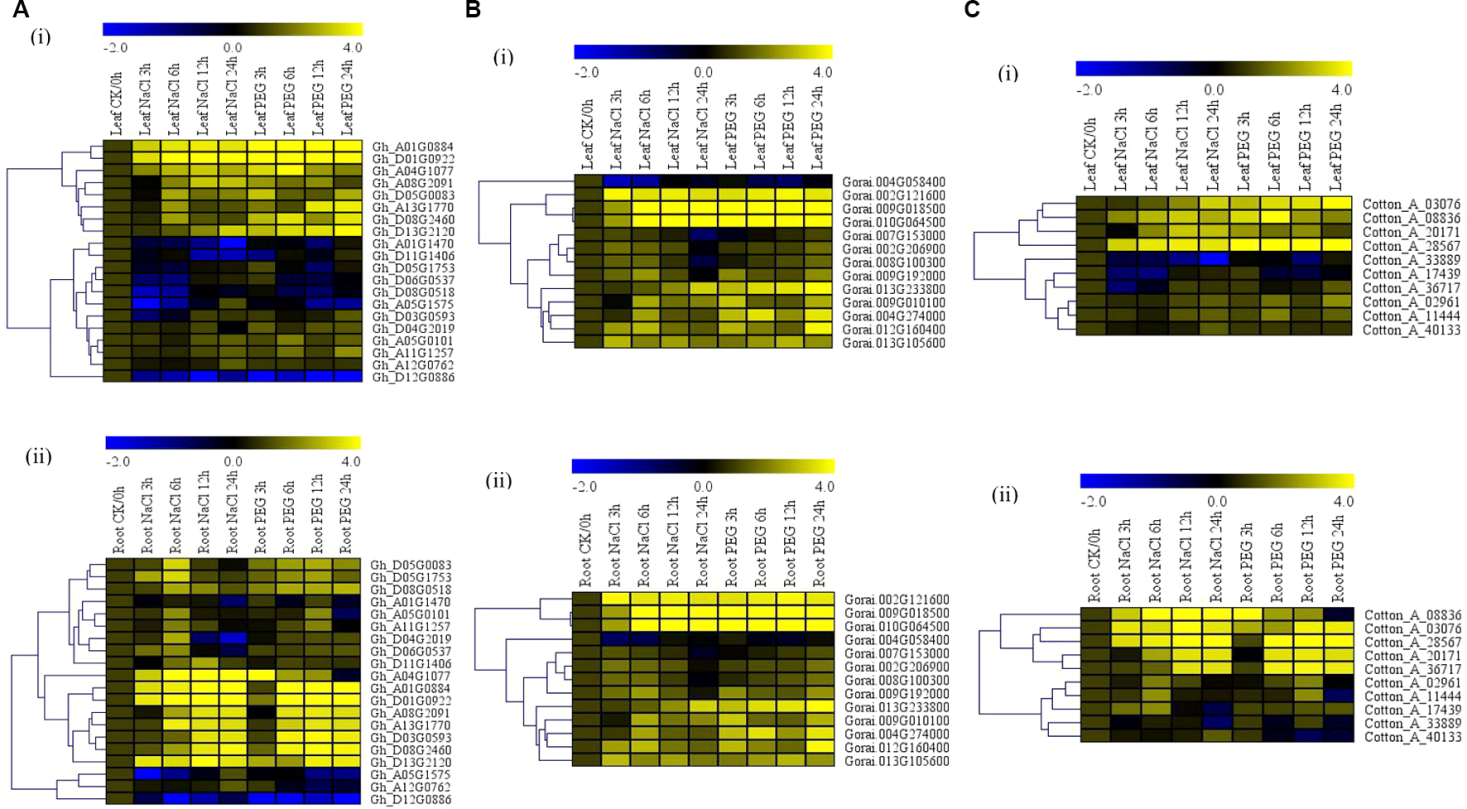
RT-qPCR analysis of the selected upland cotton *Alba* genes under water deficit and salt stress conditions. **(A**–**C)** Expression level of the selected cotton, *G. hirsutum*, *G. raimondii* and *G. arboreum* Alba genes under drought and salt stress conditions. The heatmap was visualized using MeV.exe program (showed by log 2 values). In control, and in treated samples, 1, 3, 6, and 12h poststress treatment. (i) Yellow—up-regulated, blue—down-regulated, and black—no expression.

For RT-qPCR validation, 20 (61%), 13 (65%), and 10 (59%) genes were used for *G. hirsutum*, *G. raimondii*, and *G. arboreum*, respectively. The genes were chosen as per the results obtained from the phylogenetic tree analysis, RNA expression, and gene structure analysis. Based on phylogenetic tree analysis, the proteins encoded by the cotton *Alba* genes were grouped into four clades, but clade 3 members were least, which were majorly composed of Alba proteins from other plants used for the tree analysis; so, clades 1, 2, and 4 were considered for the gene selections. In relation to gene structure, we analyzed to take into consideration the nature of intron disruption; the selected genes shared a common gene structure attribute, with intron number ranging from five to nine. Finally, the secondary RNA sequenced data was obtained from the cotton genome database (https://cottonfgd.org/analyze/); only those genes which showed significance upregulation was finally chosen for further analysis through RT-qPCR validation, just as it has been previously described by Magwanga et al. (Magwanga et al., 2018), in the analysis of the *LEA* genes in upland cotton (**Table S5**). Only two tissues root and leaf were investigated under water deficit and salt stress conditions. The *Alba* genes across the three cotton species exhibited a similar expression pattern, in which more genes were found to be significantly up-regulated in the root tissues but not in the leaf (**Table S6**). In *G. hirsutum*, two genes were found to be significantly up-regulated in the leaf under water deficit and salt stress conditions; the same expression pattern was replicated in the roots under similar stress conditions, *Gh_A01G0884 (ghAlba_4)* and *Gh_D01G0922 (ghAlba_5)* (**Figure 1A**). A unique expression pattern was noted among the highly upregulated genes; the significantly up-regulated genes were observed both in root and leaves under water deficit and salt stress conditions—for instance, *Gorai.002G121600*, *Gorai.009G018500*, and *Gorai.010G064500* were highly upregulated in root and leaf tissues (**Figure 1B**); similarly, so were *Cotton_A_28567*, *Cotton_A_20171*, *Cotton_A_08836*, and *Cotton_A_03076* (**Figure 1C**).

### The Efficiency of *Gh_A01G0884 (ghAlba_4)* and *Gh_D01G0922 (ghAlba_5)* Gene Silencing in Cotton

**Figure 2:**
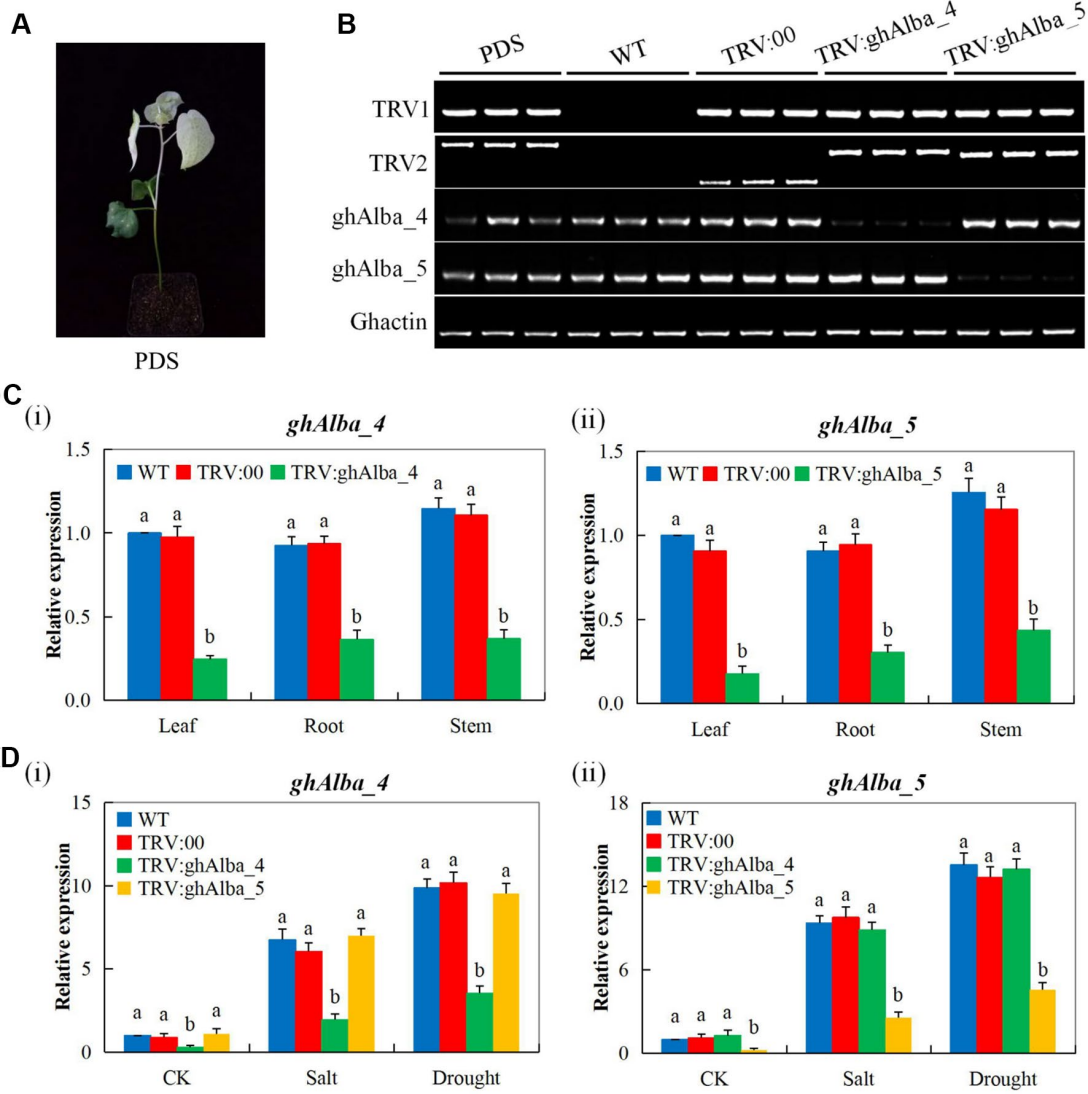
Phenotype observed in the silenced plants with the TRV2:00 empty vector, wild type plants, and *Gh_A01G0884 (ghAlba_4)*- and *Gh_D01G0922 (ghAlba_5)*-silenced plants. **(A)** Albino appearance on the leaves of the Phytoene desaturase (PDS) infused plants. **(B)** Gel electrophoresis in determining the effectiveness of gene silencing by the vector. **(C)** RT-qPCR analysis of the change in the expression level of Gh_A01G0884 (ghAlba_4) and Gh_D01G0922 (ghAlba_5) genes in cotton plants treated with VIGS. "Gh_A01G0884 (ghAlba_4) and TRV2:Gh_D01G0922 (ghAlba_5)” represent the Gh_A01G0884 (ghAlba_4) and Gh_D01G0922 (ghAlba_5)-silenced plants. **(D)** Gel electrophoresis in determining the effectiveness of gene silencing by the vector. The letters a/b indicate statistically significant differences (two-tailed, *p < 0.05*) between the samples in each treatment. Error bars of the *Gh_A01G0884 (ghAlba_4)* and *Gh_D01G0922 (ghAlba_5)* gene expression levels represent the standard deviation of three biological replicates.

The albino trait was observed among the plants infused with the phytoene desaturase gene (TRV-PDS) after 12 days of post-inoculation (dpi). The leaves and the stem region above the cotyledon became chlorotic thus exhibited the albino type of characteristics while the VIGS, wild types, and the positively controlled plants showed normal leaf color (**Figure 2A**). Moreover, the knockdown of the two *Alba* genes was further confirmed through carrying out a half RT-qPCR from the RNAs extracted from the PDS infused plants wild type, the positively controlled plants, and the two VIGS-plants using their specific primers. The TRV1 and 2 bands were never detected on the WT plants, but bands were formed in TRV:00, PDS, TRV: Alba_4, and TRV:Alba_5 infused plants; similarly, the two knocked genes bands were amplified in PDS, WT, and TRV:00 but showed thin bands on either of their extracted RNAs (**Figure 2B**). The bands were checked with an internal control gene, *GhActin*. These results showed that the targeted genes were effectively knocked down in the cotton plants. The efficiency of the VIGS on the plants is monitored phenotypically by the albino-like appearance on the leaves (Gao et al., 2011b). To further determine the efficiency level of the gene knockdown through VIGS, RT-qPCR assay was carried out on the leaf, stem, and root tissues collected from the TRV:*Gh_A01G0884 (ghAlba_4)* and TRV:*Gh_D01G0922 (ghAlba_5)* constructs, wild type, and the positively controlled plants. The transcript expression levels of the knocked genes, Gh_*A01G0884 (ghAlba_4)* and *Gh_D01G0922 (ghAlba_5)*, were significantly reduced in the *Gh_A01G0884 (ghAlba_4)*- and *Gh_D01G0922 (ghAlba_5)*-silenced plants compared with their expression levels in the wild type and the positive control plants; though in the VIGS-plants, the expression levels of the knocked genes were relatively higher in the leaves compared to other tissues, such as the stem and the roots (**Figures 2C–D**).

### Physiological Traits Evaluation and Root Assays of the VIGS Plants and the Wild Types Under Water Deficit and Salt Stress Conditions

**Figure 3:**
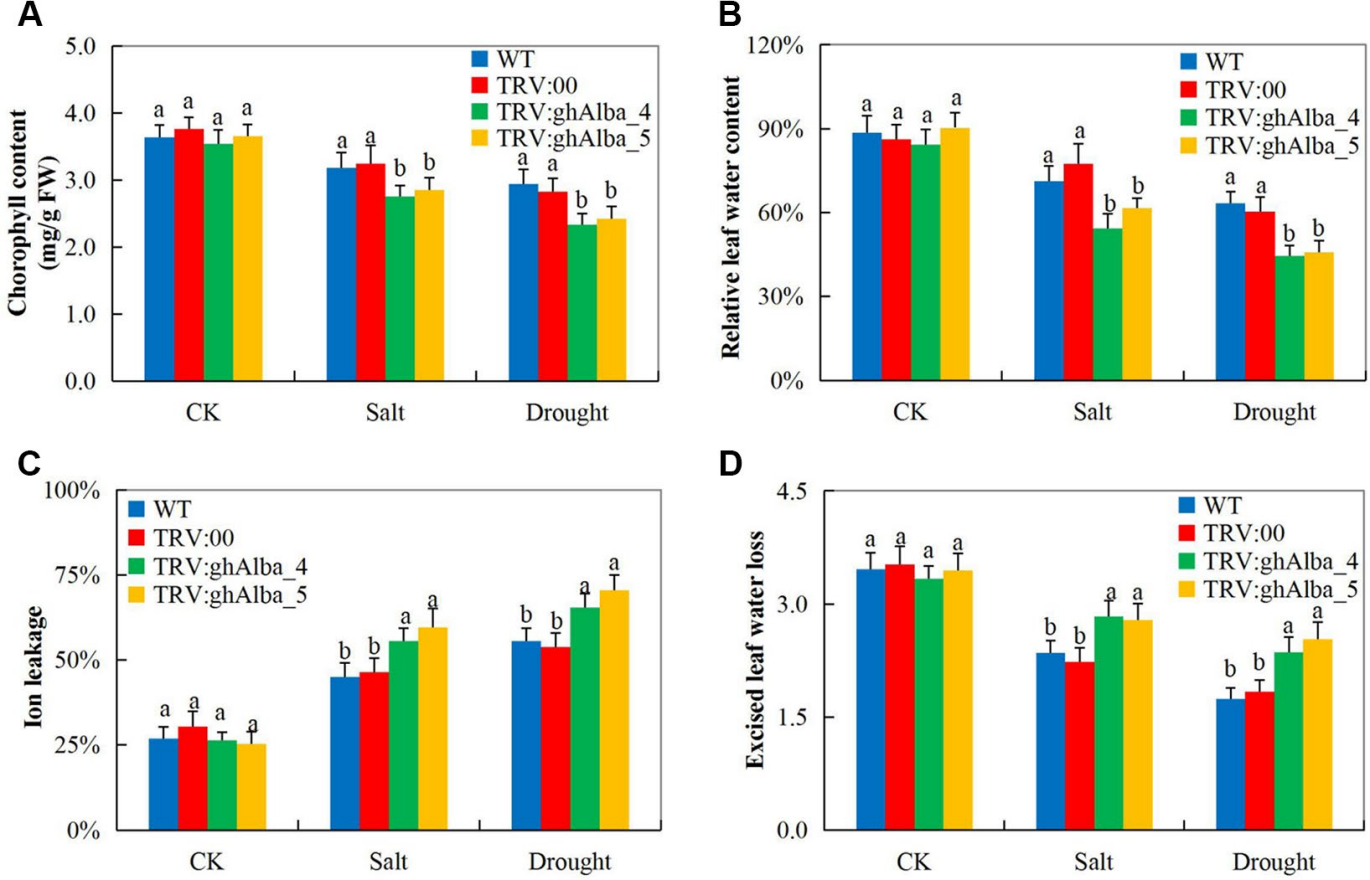
Evaluation of physiological traits in the VIGS-treated plants. **(A)** Chlorophyll content in the TRV2 empty vector-carrying plants and *Gh_A01G0884 (ghAlba_4)*- and *Gh_D01G0922 (ghAlba_5)*-silenced plants. **(B)** The excised leaf water loss (ELWL) level in the TRV2 empty vector-carrying plants and *Gh_A01G0884 (ghAlba_4)*- and *Gh_D01G0922 (ghAlba_5)*-silenced plants. **(C)** The relative leaf water content (RLWC) in the TRV2 empty vector-carrying plants and *Gh_A01G0884 (ghAlba_4)*- and *Gh_D01G0922 (ghAlba_5)*-silenced plants. **(D)** The cell membrane stability (CMS) evaluated through ion leakage in the TRV2 empty vector-carrying plants and *Gh_A01G0884 (ghAlba_4)*- and *Gh_D01G0922 (ghAlba_5)*-silenced plants. The letters a/b indicated statistically significant differences (two-tailed, *p < 0.05*) between the samples in each treatment. Error bars represent the standard deviation of three biological replicates.

Evaluation of the physiological traits, the *Gh_A01G0884 (ghAlba_4)*- and *Gh_D01G0922 (ghAlba_5)*-silenced plants showed a significant reduction in CMS, ELWL, chlorophyll content, and RLWC compared with the wild types and the positive control plants (**Figures 3A–D**). The reduction in CMS as evident by high ion leakage showed that *Gh_A01G0884 (ghAlba_4)*- and *Gh_D01G0922 (ghAlba_5)*-silenced plants suffered more of oxidative stress, and their membrane integrity was highly affected; the results were coherent with previous findings in which the knockdown of trihelix (TH), the plant TFs, affected the CMS and, in turn, increased the level of ion leakage (Magwanga et al., 2019a). Under environmental stress condition, the plant’s inability to assimilate sufficient amount of carbon (IV) oxide lead to increased photorespiration, thus higher production of hydrogen peroxide (Choudhury et al., 2017). Excess accumulation of reactive oxygen species (ROS) does cause cellular damage, which eventually leads to plant death (Mirza et al., 2013).

**Figure 4:**
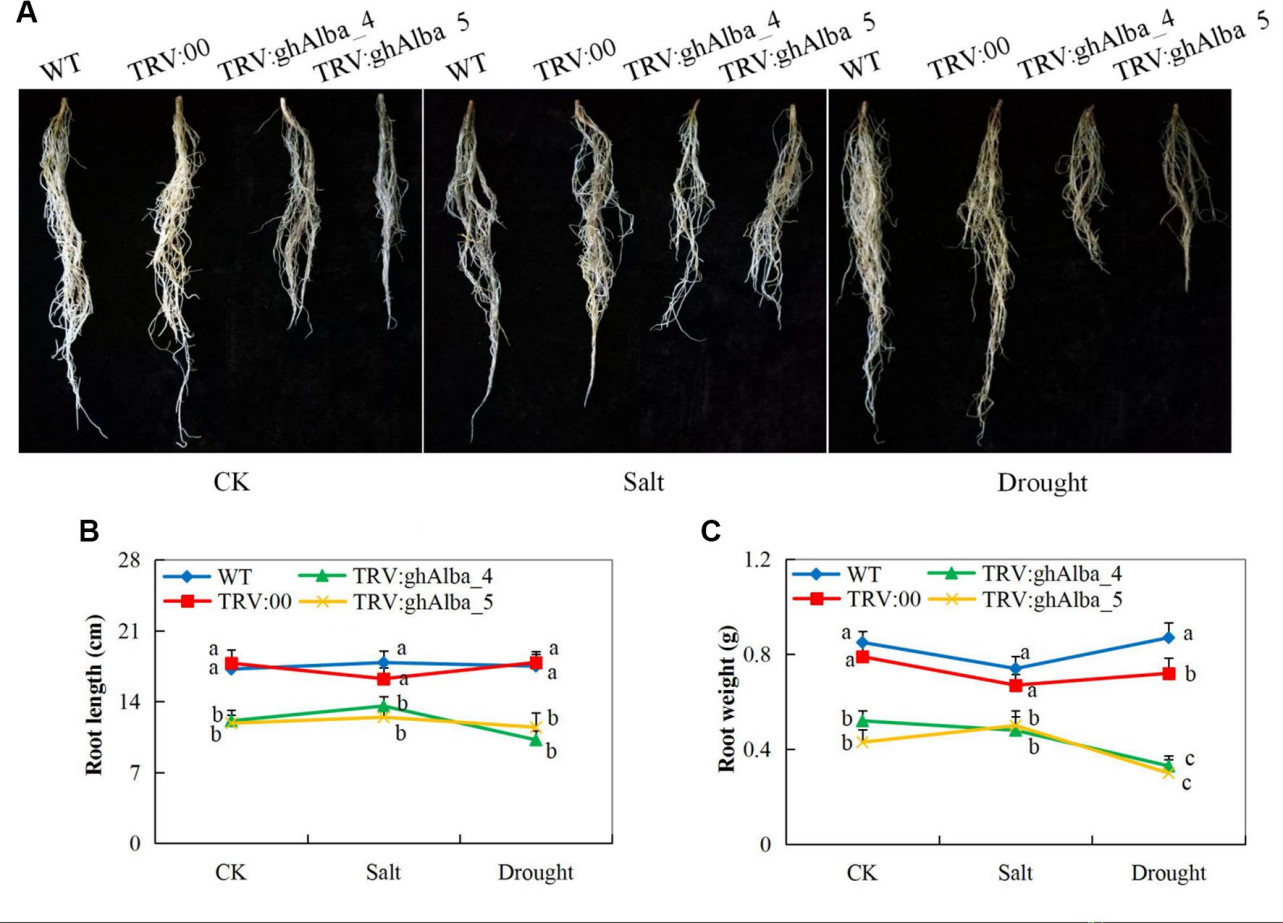
Root evaluation in the VIGS-treated plants **(A)** Root length in the TRV2 empty vector-carrying plants and Gh_A01G0884 (ghAlba_4) and Gh_D01G0922 (ghAlba_5)-silenced plants, photographs taken after 8 days of stress exposure **(B)** Statistical determination of the root length in the TRV2 empty vector-carrying plants and Gh_A01G0884 (ghAlba_4) and Gh_D01G0922 (ghAlba_5)-silenced plants **(C)** Root weight measurements in the TRV2 empty vector-carrying plants and Gh_A01G0884 (ghAlba_4) and Gh_D01G0922 (ghAlba_5)-silenced plants. The letters a,b,c indicated statistically significant differences (two-tailed, p < 0.05) between the samples in each treatment. Error bars represent the standard deviation of three biological replicates.

The two cotton plants showed significant variation in root lengths and biomass accumulation. The *Gh_A01G0884 (ghAlba_4)*- and *Gh_D01G0922 (ghAlba_5)*-silenced plants under treatment and control conditions compared with the pTRV2 (empty vector) were infused, and wild types had reduced root growth with relatively low emergence of lateral roots (**Figures 4A–C**). The results showed that the silencing of the *Alba* genes had a negative effect on root growth. Genes have been found to have a dominant effect on plant root growth—for instance, overexpression of water deficit inducible *OsERF48* gene has been found to regulate rice calmodulin-like protein (*OsCML16*) gene, which promotes plant root growth and in turn enhance water deficit tolerance (Jung et al., 2017). The root is an important organ; it contributes directly to crop performance (Rogers and Benfey, 2015) and is the primary organ for the uptake of water and nutrients, which are the raw materials for photosynthesis in plants (Yamauchi et al., 2018). We hypothesize that the downregulation of the *Alba* genes could have an effect on the quiescent center on the root primordial region, thereby lowering the rate of cell division, enlargement, and elongation, which are the main cellular processes contributing to root growth.

### The Oxidant, Antioxidant, and Abiotic Stress-Responsive Evaluation Between the VIGS and the Wild Cotton Varieties Under Water Deficit and Salt Stress Conditions

**Figure 5:**
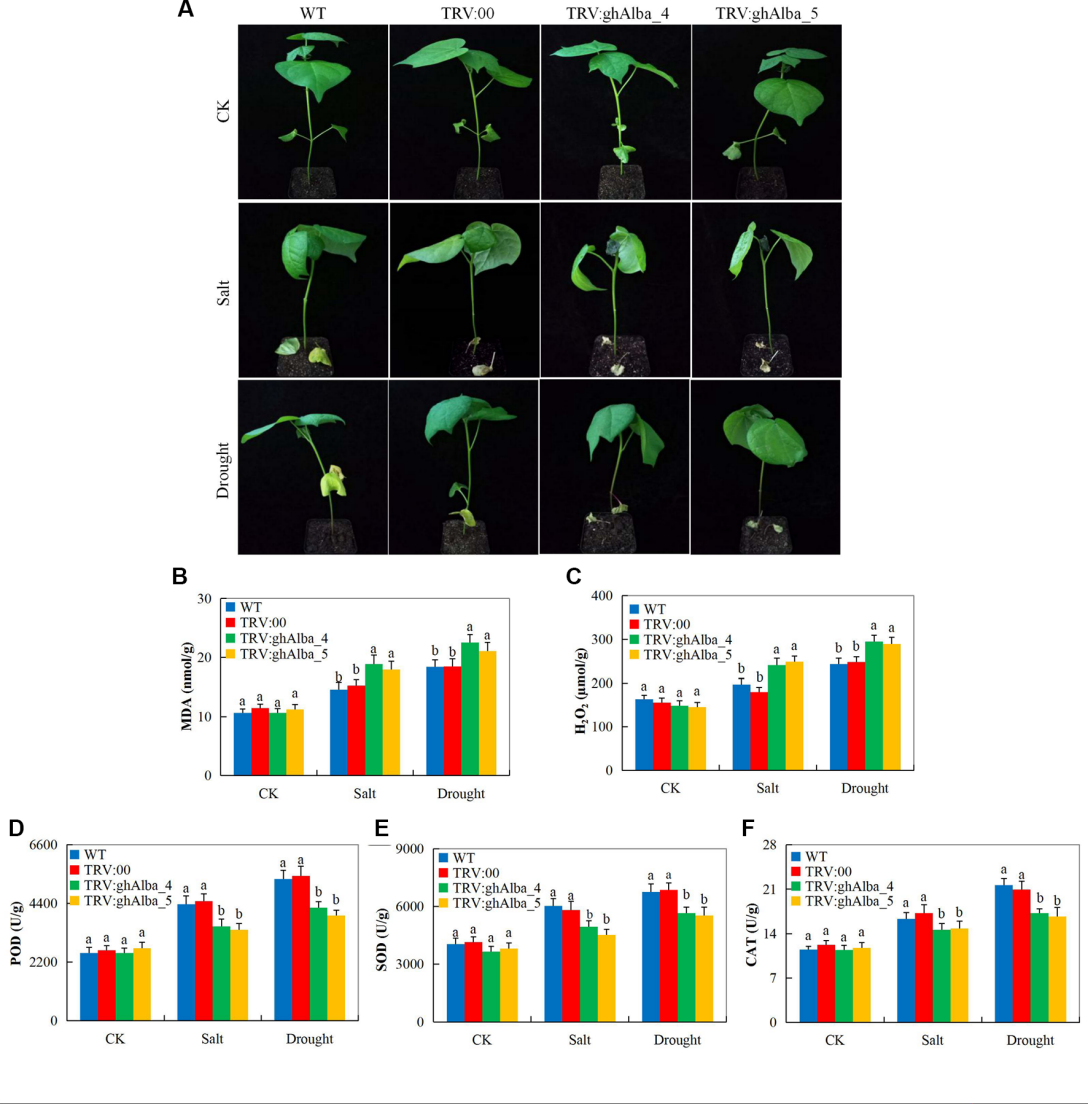
Phenotypic observation and the determination levels of the accumulation of oxidants and antioxidant enzymes in the VIGS-treated plants. **(A)** The phenotypic observation on the VIGS and wild types after drought and salt stress treatments, drought imposed by withdrawal of watering for 6 days while salt stress was initiated by irrigating the plants with 250 mM of NaCl solution for a period of 4 days. **(B**–**F**) The H_2_O_2_, POD, SOD, and CAT contents in the TRV2 empty vector-carrying plants and *Gh_A01G0884 (ghAlba_4)*- and *Gh_D01G0922 (ghAlba_5)*-silenced plants. The letters a/b indicated statistically significant differences (two-tailed, *p < 0.05*) between the samples in each treatment set. Error bars of the H_2_O_2_, POD, SOD, and CAT contents represent the standard deviation of three biological replicates.

Evaluation of the oxidants and antioxidant enzymes showed that *Gh_A01G0884 (ghAlba_4)*- and *Gh_D01G0922 (ghAlba_5)*-silenced plants were highly affected under water deficit and salt stress compared with the wild types and the control plants. The VIGS plants showed drought and salt stress symptoms on their leaves compared to the wild types (**Figure 5**), but when the positively controlled, the wild types, and the VIGS plants under drought stress were re-watered for a period of 3 days, the positively controlled and the wild type plants showed a significantly higher level of recovery compared to the VIGS plants (**Figure S5A**). The survival rate of the wilt type, the positively controlled, and the VIGS plants were 55% (11 of 20 plants), 50% (10 of 20 plants), and 20% (5 of 20 plants), respectively (**Figure S5B**). The results were in agreement to the finding obtained when the *SpMPK1*, *SpMPK2*, and *SpMPK3* were knocked down in tomato; the survival rate of the VIGS plants was significantly lower than the wild types (Li et al., 2013). Moreover, evaluation of the oxidant and antioxidant enzymes such as POD, SOD, CAT, MDA, and H_2_O_2_ revealed that the VIGS-plants were significantly affected under drought and salt stress conditions compared to the wild types. The VIGS-plants and the wild types exhibited no significance difference under controlled conditions in all the biochemical parameters evaluated; however, under drought and salt stress conditions, MDA and H_2_O_2_ were significantly higher in concentration in the leaves of the VIGS plants but lower in the wild types (**Figures 5B–C**). Furthermore, three antioxidant enzymes were assayed; POD, SOD, and CAT all registered significant reduction on the leaves of VIGS plants under drought and salt stress, while there were no significant differences observed in their levels on the VIGS and wild types under controlled conditions (**Figures 5D–F**). The results obtained were in agreement to previous findings in which plants which are susceptible to any form of abiotic stress factor do register higher levels of oxidant enzymes as opposed to antioxidant under stress (Lu et al., 2018b).When plants are exposed to either abiotic or biotic stress conditions, the normal balance between ROS production and elimination shifts, leading to excessive accumulation of ROS and, in turn, resulting in massive oxidative damage, causing extensive cellular damage and inhibition of photosynthesis which limit the plant productivity. The excess ROS is then catalyzed into non-destructive form by antioxidant enzymes, such as catalase (CAT), peroxidase (POD) and superoxide dismutase (SOD), ascorbate peroxidase (APX), and polyphenoloxidase (PPO), among others (Wang et al., 2017a). The SOD is the first enzyme involved in the detoxification of ROS and converts superoxide (O_2_^−^) radicals to H_2_O_2_ (Kuo et al., 2013). The significant reduction in the concentration levels of the various antioxidant enzymes evaluated showed that the *Gh_A01G0884 (ghAlba_4)*- and *Gh_D01G0922 (ghAlba_5)*-silenced plants were highly susceptible to drought and salt stresses compared with to the control and wild types, an indication showing that the *Alba* genes are integral in enhancing abiotic stress tolerance in plants.

### Transcription Analysis of Abiotic Stress-Responsive Genes on the Tissues of VIGS-Plants and Wild Types Under Drought and Salt Stress Conditions

**Figure 6:**
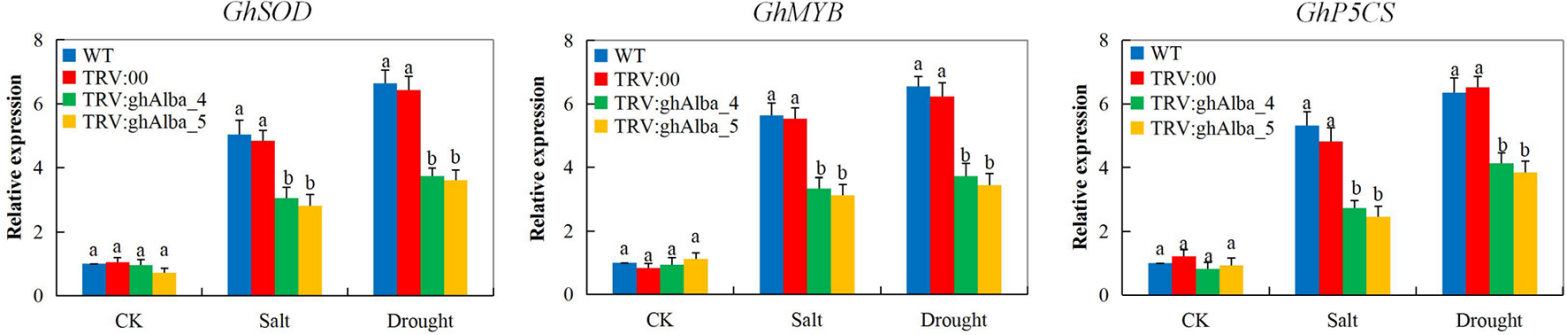
RT-qPCR analysis of the change in the expression levels of the *GhP5CS, GhMYB, and GhSOD* stress response genes in cotton plants treated with VIGS. “TRV2:00” represents the plants carrying control the TRV2 empty vector; “TRV2:*Gh_A01G0884 (ghAlba_4)* and TRV2:*Gh_D01G0922 (ghAlba_5)*” represent the *Gh_A01G0884 (ghAlba_4)*- and *Gh_D01G0922 (ghAlba_5)*-silenced plants. The letters a/b indicate statistically significant differences (two-tailed, *p < 0.05*) between the samples in each treatment. Error bars of the gene expression levels represent the standard deviation of three biological replicates.

The ability of the plants to induct stress-responsive genes indicates their ability to tolerate the stress levels (Jorge et al., 2017). In the evaluation of three abiotic stress-responsive genes, cotton superoxide dismutase (*GhSOD*), cotton myeloblastosis (*GhMYB*), and cotton delta-1-pyrroline-5-carboxylate synthetase (*GhP5CS*) showed the knockdown of the two *Alba* genes, *ghAlba_4* and *ghAlba_5*, significantly affected the ability of the VIGS plants to induce more stress-responsive genes in order to improve their ability to tolerate the effects caused by drought and salt stresses. The expression levels of all the genes showed significant downregulation in the VIGS plants compared to their wild types under drought and salt stress conditions; however, under normal condition, no significant variation was observed among the VIGS and the wild types, an indication that the stress-responsive genes are only induced by the plants under stress conditions (**Figure 6**). The results obtained were in agreement to the previous finding in which the knockdown of cotton *CYP450* genes significantly affected the ability of the plants to tolerate drought and or salt stress, and thus *GhSOD, GhP5CS*, and *GhMYB* genes were all downregulated in the VIGS plants (Magwanga et al., 2019b). The first line of defense by plants against the deleterious effects of ROS due to abiotic stresses is the SODs which convert O_2_^−^ into H_2_O_2_; this is because of its presence in all the cellular compartments (Balamurugan et al., 2018). Furthermore, the MYB is among the top-ranked stress-responsive plant’s TFs together with NAC family members, and thus a number of investigations have revealed the key regulatory roles played by the MYBs in plant growth, development, and abiotic stress response (Tang et al., 2019). Moreover, delta-1-pyrroline-5-carboxylate synthase genes have been demonstrated to be vital in the proline biosynthesis pathways and are significantly induced by drought stress (Xia et al., 2017). The ability of the plants to induct the Alba proteins encoded by the *Alba* genes enables the plants to maintain the photosynthetic process and other drought and or salinity stress related tolerant mechanisms thus enhances the plants survival under drought and salt stress conditions (**Figure S6**). Thus, the downregulation of these stress-responsive genes showed that the knockdown of the *Alba* genes significantly reduced the tolerance levels of the cotton plants to drought and salt stresses.

## Discussion

Unfavorable environmental changes have become reality, and this is projected to worsen if the rate of environmental degradation is not abated (Yadav et al., 2018). Plants being poikilothermic and sessile in nature, erratic environmental changes, do result in major losses in both yield and quality of the products (Halford et al., 2015). Non-edible plant with bushy architecture is a challenge to be grown under greenhouse conditions; thus, a number of crops are mainly grown in the fields, more so cotton, due to mechanization requirement and long growth periods. Several studies have been carried out in order to investigate the effects of abiotic stress on cotton production, and it has been shown that drought, salt, and extreme temperature stress are the major factors hindering full potential in cotton production (Isoda, 2010; Nachimuthu and Webb, 2017). Due to extreme growing conditions, plants have evolved various adaptive strategies at morphological, physiological, and molecular levels in order to reduce the effects of different abiotic stress factors (Rasool et al., 2019). At the molecular level, several stress-responsive genes and plant TFs have undergone tremendous transitions. Plants are capable to induct more of the genes and TFs to enable themselves to tolerate various stress factors. One of the gene families highly associated with stress tolerance is *Alba* genes (Kumar Verma et al., 2018). In this study, we identified various *Alba* genes in the three cotton species, *G. hirsutum*, *G. arboreum*, and *G. raimondii*, and found 33, 17, and 20 genes, respectively. The number of genes detected is in line with the nature of the three cotton genomes; *G. hirsutum* is a tetraploid cotton (AADD), having emerged through WGD of both the *G. arboreum* (AA) and *G. raimondii* (DD) (Yu-xiang et al., 2013); thus, the high number of genes in *G. hirsutum* affirms this evolution process. Some level of gene loss or duplication was detected; the numbers of genes in tetraploid cotton were less than the exact the number of proteins encoded by the *Alba* genes in the two diploid cotton parents. If the principle of WGD was to hold, it therefore means that either the tetraploid *Alba* genes lost some function or either of the two diploid *Alba* genes underwent duplication over time in the course of their evolution; though, this needs further investigation.

RNA expression profiling and RT-qPCR validation of the genes under water deficit and salt stresses showed that major genes were highly induced in the root tissues compared to other organs. The roots are the primary tissues which bear the full effect of water deficit and or salt stress being into contact with dry soil in case of water deficit or saline soil for the salt stress condition (Kunert et al., 2016). The highly upregulated genes in the root tissues were also found to be targeted by specific miRNAs—for instance, *Gh_D01G0922* was target by ghr-miR396a and ghr-miR396b; the same miRNA has been found to be highly expressed in *Arabidopsis* (Liu et al., 2008), *Zea mays* (Ding et al., 2009), *O. sativa* (Gao et al., 2011a), and *G. max* (Li et al., 2011) under salt and water deficit conditions. In addition, miR396b has been found to exhibit at least two-fold changes under water deficit only in *CB46*, a drought-sensitive cowpea genotype (Barrera-Figueroa et al., 2011). Moreover, the highly up-regulated genes within the root tissues under water deficit and salt stresses were also found to be associated with some unique *cis*-regulatory elements—for instance, ABRELATERD1 (ACGTG )with a role in early responsive to dehydration, ARFAT (TGTCTC) in dehydration-responsiveness, CBFHV (RYCGAC) for dehydration-responsive element (DRE)/low temperature, LTRE1HVBLT49 (CCGAAA) as low-temperature-responsive element, MYBCORE (CNGTTR) in dehydration/water stress, and MYCCONSENSUSAT (CANNTG) as dehydration-responsive gene/cold stress. Similar *cis*-regulatory elements have been found to regulate some of the stress-responsive genes, such as the *LEA* genes (Magwanga et al., 2018).

In the functional characterization of the two highly upregulated *Alba* genes under water deficit and salt stress conditions, we carried out VIGS of the two genes, *Gh_A01G0884 (ghAlba_4)* and *Gh_D01G0922 (ghAlba_5)*; in upland cotton, the VIGS and the wild type were exposed to salt and water deficit conditions. The VIGS cotton genotype was highly affected by water deficit and salt stress compared to the wild type. Chlorophyll content, CMS, saturated leaf weight (SLW), ELWL, and root traits showed negative deviation compared to the wild type, indicating that the wild type had a higher capacity to tolerate the effects caused by water deficit and salt stresses. Similarly, analysis of the reactive oxygen scavenging species, the antioxidant enzymes, POD, SOD, and CAT was significantly reduced in the leaves of the VIGS cotton. Moreover, the evaluation of the oxidants showed that H_2_O_2_ and MDA concentrations were significantly higher in the leaves of the VIGS than the wild type. When plants are exposed to any stress, the equilibrium between ROS release and detoxification becomes altered, thus leading to higher accumulation of ROS. Excess ROS results in oxidative injuries, which eventually lead to plant death. The low ROS scavenging enzymes in the leaves of the VIGS exhibited higher oxidative injuries compared to the wild type.

The SOD does constitute the first line of the plant’s defense against the deleterious effects of the ROS when plants are exposed to any form of abiotic stress; the ROS production increases leading to excessive accumulation (Sharma et al., 2012). In plants, O^2−^ is produced at any cellular sites as long as the electron transport chain is involved (Grene, 2002); thus, O_2_ activation is likely to occur in plant cellular structures such as the mitochondria microsomes, glyoxysomes, peroxisomes, chloroplasts, cytosol, and the apoplasts (Elstner and Osswald, 1994). Thus, the level of ROS is checked by the activation of the antioxidant enzymes such as the SOD; thus, the lower concentration of this protein encoded by the *GhSOD* genes within the leaves of the VIGS cotton showed that the plants ability to regulate the amount of ROS was highly affected and thus were subjected to oxidative damage as a result of salt and water deficit exposure. In addition, the expression of the *GhMYB* gene was significantly downregulated in the VIGS cotton compared to the wild types. The plant exposure to various stress factors triggers a well-coordinated changes in gene expression (Virlouvet et al., 2018); the MYBs are among the top-ranked plants TFs highly associated with significant roles in promoting plants tolerance to various abiotic stress factors (Wang et al., 2017b), but it is worth noting that the genes work in a synchronized manner, the downregulation of the two *Alba* genes, affected the expression levels of the MYBs. Moreover, the pyrroline-5-carboxylate synthase (P5CS) enzyme is critical in catalyzing the various reaction leading to proline biosynthesis, and proline has been found to have a protective role against environmental and non-environmental stress effects in plants (Rai and Penna, 2013).

The proteins encoded by the Alba genes have an integral role in the genome construction of an organism and in turn control the expression dynamics of a number of genes in the organisms (Kumar Verma et al., 2018). Moreover, characterization of *Arabidopsis* *Alba* genes, *AtALBA1* and *AtALBA2*, revealed that, despite their differences in nucleic acid binding properties, they are located within the localized within the nucleus and mainly form a heterodimers in the nucleus and do bind the R-loop structures, and their depletion results in hypersensitivity of the plants to DNA damaging agents as a result of abiotic stress factors (Yuan et al., 2019). The heterodimers are vital in immune response—for instance, in rice, OsCERK1 forms a heterodimer complex with OsCEBiP, which is a LysM-containing receptor-like protein and directly binds chitin, to induce immune responses (Kouzai et al., 2014). The downregulation of the cotton *GhP5CS* gene in the tissues of the VIGS cotton indicated that the proline biosynthesis cycle is altered, and plant ability to tolerate salt and water deficit was highly compromised. Moreover, the increased levels of the oxidant enzymes, such as the MDA and H_2_O_2_, showed that the knockdown of *ghAlba_4* and *ghAlba_5* significantly affected the ability of the plants to tolerate the effects of drought and salt stresses and a thus higher level of oxidative.

## Conclusion

In conclusion, the identification and functional characterization of the *Alba* proteins in upland cotton provide fundamental information for future exploration of this diverse and yet underexplored plant protein family. This study gives the very first insight evaluation of the proteins encoded by the *Alba* genes in cotton. A total of 33, 20, and 17 proteins encoded by the *Alba* genes were identified in *G. hirsutum, G. raimondii*, and *G. arboreum*, respectively. The total number of the Alba proteins in the two diploid cottons, *G. raimondii* of the D genome and *G. arboreum* of the A genome, is less than the number of Alba proteins obtained for the tetraploid cotton *G. hirsutum*, even though the tetraploid cotton emerged as a result of WGD of the A and D (Lee and Fang, 2015), The low number could be attributed to gene loss after the emergence of the tetraploid cotton. The virus gene silencing (VIGS) of the two novel *Alba* genes in cotton revealed that the proteins encoded by the *Alba* genes are critical in enhancing root growth; primary growth is an important trait in xerophytic plants; and long and widely extended roots increase the rate of water absorption, thus improving the drought responsible mechanism among the xerophytic plants (Moriuchi and Winn, 2005). Furthermore, the VIGS plants when subjected to osmotic and salt stresses were found to have higher levels of the oxidant and significant reduction in antioxidant enzymes such as CAT, POD, and SOD, an indication that the seedlings were under intense oxidative stress compared to their wild types under similar conditions. Moreover, known stress-responsive genes such as *GhSOD, GhMYB*, and *GhP5CS* were all downregulated in the tissues of the VIGS cotton but were significantly upregulated in wild types under water deficit and salt stress conditions, which further augmented our results, in the validation of the possible roles of the proteins encoded by the *Alba* genes in enhancing water deficit and salt stress tolerance in cotton. We hereby propose further research to explore the exact role of the proteins encoded by the *Alba* genes at the cellular level.

## Data Availability Statement

All datasets generated for this study are included in the manuscript/**Supplementary Files**.

## Author Contributions

RM and FL designed the experiment, RM, PL and JK implemented and collected the data. RM analyzed the results and prepared the manuscript. RM, JK, PL, SA, FL, XW, XC, ZZ, YX, YH, KW and FL revised the manuscript. All authors reviewed and approved the final manuscript.

## Funding

The research work was funded by the National key research and development plan (2016YFD0100306) and the National Natural Science Foundation of China (31671745, 31530053).

## Conflict of Interest

The authors declare that the research was conducted in the absence of any commercial or financial relationships that could be construed as a potential conflict of interest.

## Supplementary Material

Figure S1 Phylogenetic tree analysis **(B)**: protein sequence alignment of the Alba Genes in *G. hirsutum*, *G. raimondii*, *G. arboreum*, *Oryza sativa*, *Theobroma cacao*, *Arabidopsis thaliana*, *Sorghum bicolor*, *Populus trichocarpa* and *Glycine max*. The red enclosure indicates the cloned genes.

Figure S2 Cotton Alba gene structure and motif analysis **(A)** Phylogenetic tree, gene structure, and motif of all the *G. hirsutum Alba genes*. **(B)**: Phylogenetic tree analysis, gene structure, and motif identification of the proteins encoded by the Alba genes for *G. raimondii*. **(C)**: Phylogenetic tree, gene structure, and motif identification of the proteins encoded by the Alba genes for *G. arboreum*. Red: exons, blue: up/downstream, and the grey lines: the introns.

Figure S3 Average number of the cis-regulatory elements in the promoter region of the three cotton species Alba genes. The cis-regulatory elements were analyzed in the 1 kb up/ down stream promoter region of the translation start site using the online tool, PLACE database.

Figure S4 RNA sequence analysis of the Alba genes under water deficit and salt stress conditions. The heat map was visualized using the MeV4.9.0 program. Yellow indicates upregulation; blue indicates downregulation and black indicates no expression. **(A)** Heat map showing gene expression under salt stress conditions. **(B)** Heat map showing gene expression under water deficit condition. The abbreviations Rt: root; St: stem; Lf: leaf; CK: control condition; PEG: Polyethylene glycol for initiating osmotic stress. The RNA sequence analysis was profiled at 0h, 1h, 3h, 6h and 12h of stress exposure under salt and osmotic stress conditions.

Figure S5 Phenotype.

Table S1 Protein sequences for the Alba proteins used in the phylogenetic tree analysis. Gh: *G. hirsutum*; Gorai: *G. raimondii*, Cotton_A: *G. arboreum*; Thecca: *Theobroma cacao*, AT: *Arabidopsis thaliana*, Sobic: *S. bicolor*, Glyma: *G. max*, Potri: *P. trichorcarpa* and LOC_Os: *Oryza sativa*.

Table S2 Details of the primers used for RT-qPCR analysis.

Table S3 The predicted miRNAs found to target various cotton Alba genes cis-*Alba*.

Table S4 Detailed information on the various cis-regulatory elements found to be associated with *Alba* genes in the three sequenced cotton species.

Table S5 RNA sequence data for the upland cotton Alba genes profiled under drought and salt stress conditions.

Table S6 The RT-qPCR results for the expression profiling of the selected Alba genes for *G. hirsutum*, *G. raimondii* and *G. arboreum*. A: The leaf tissues and B: The root tissues. The 2-**△△**CT values were transformed into log 2. CK: control, NaCl: sodium chloride (salt stress), PEG: polyethylene glycol (drought stress); the leaf and root tissues were collected at 0h, 3h, 6h, 12h and 24h of stress exposure.
