# Supplementary figures and images for: Knockdown of ghAlba_4 and ghAlba_5 Proteins in Cotton Inhibits Root Growth and Increases Sensitivity to Drought and Salt Stresses
Source: Front Plant Sci. 2019 Oct 15;10:1292. doi: 10.3389/fpls.2019.01292 (PMC6804553; doi:10.3389/fpls.2019.01292)

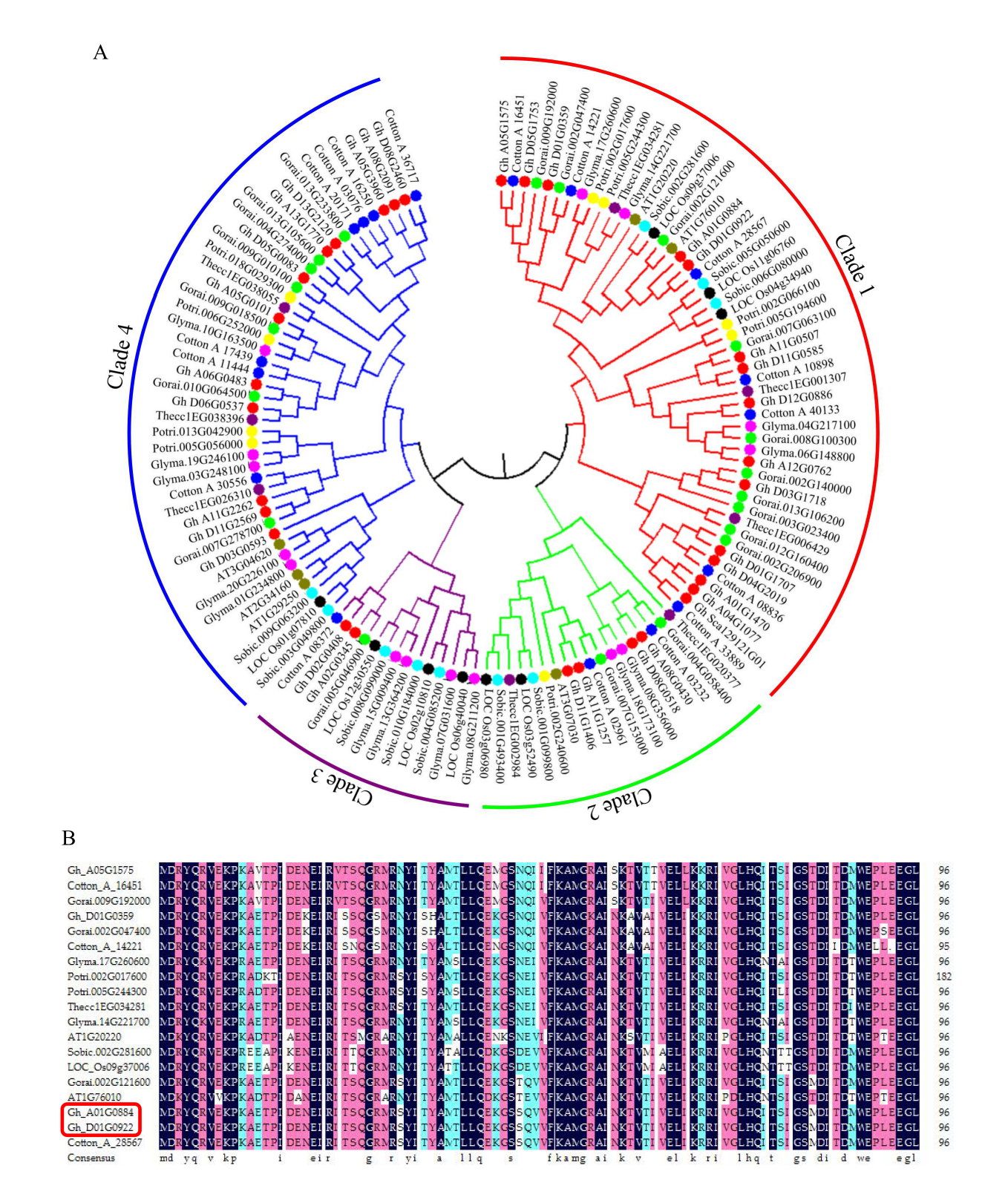

Supplement: Figure S1 — Phylogenetic tree analysis (B): protein sequence alignment of the Alba Genes in G. hirsutum, G. raimondii, G. arboreum, Oryza sativa, Theobroma cacao, Arabidopsis thaliana, Sorghum bicolor, Populus trichocarpa and Glycine max. The red enclosure indicates the cloned genes. [file Image_1.tif]

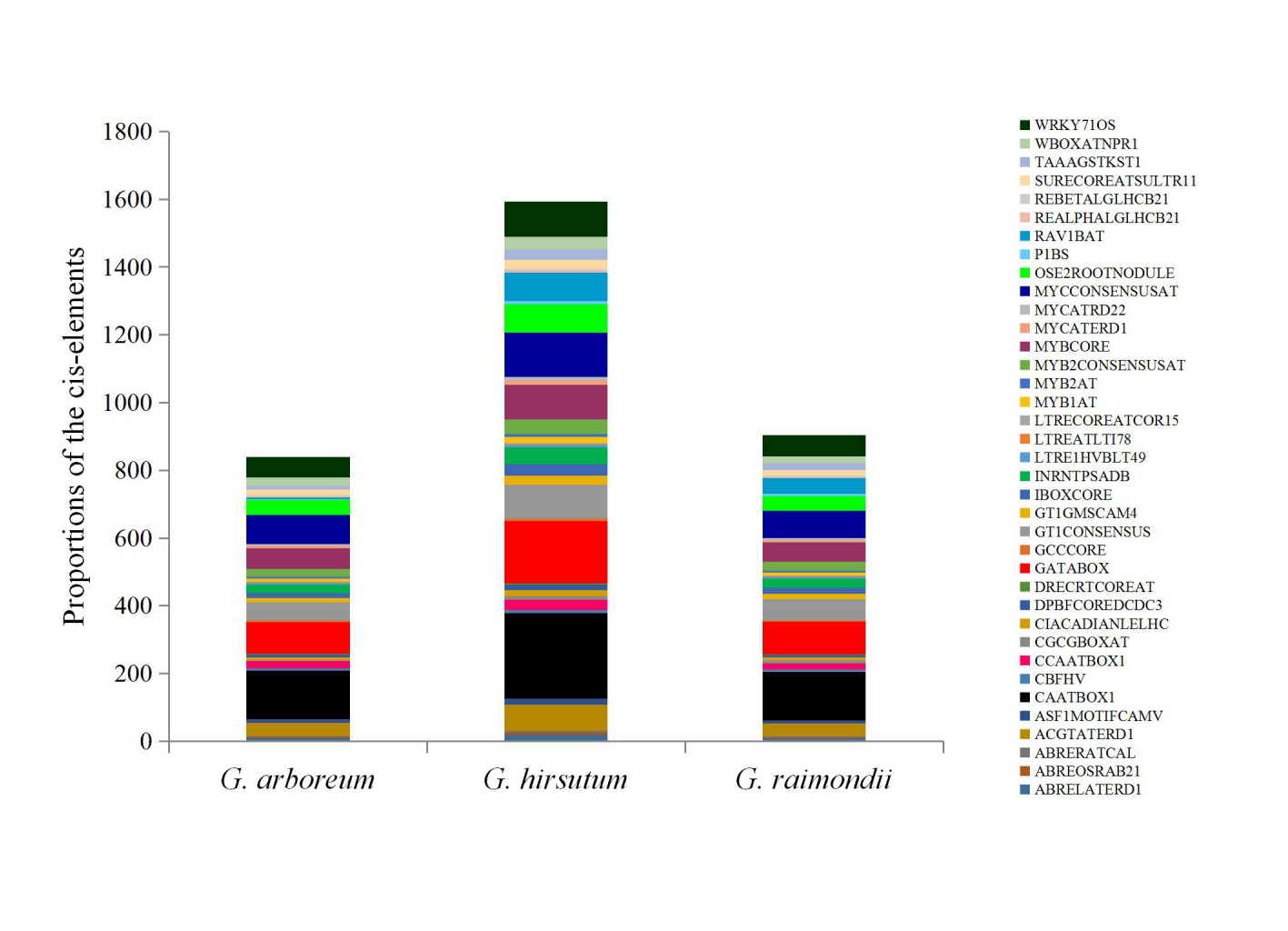

Supplement: Figure S3 — Average number of the cis-regulatory elements in the promoter region of the three cotton species Alba genes. The cis-regulatory elements were analyzed in the 1 kb up/ down stream promoter region of the translation start site using the online tool, PLACE database. [file Image_3.tif]

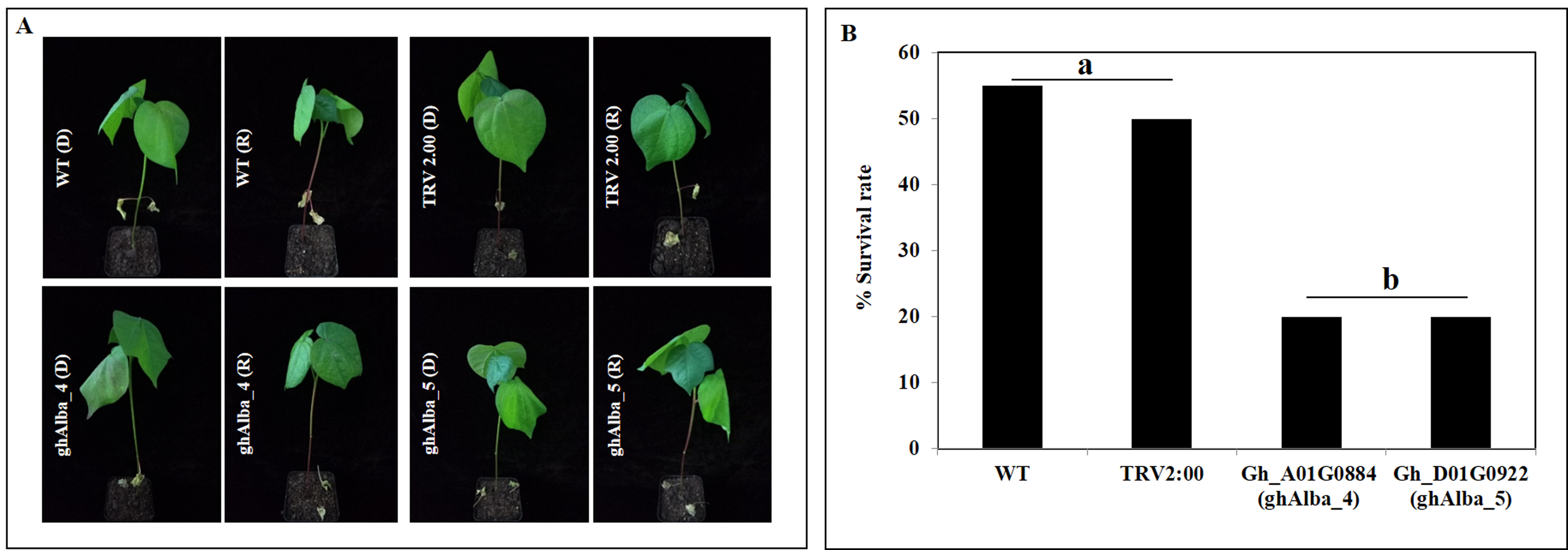

Supplement: Figure S5 — Phenotype. [file Image_5.tif]

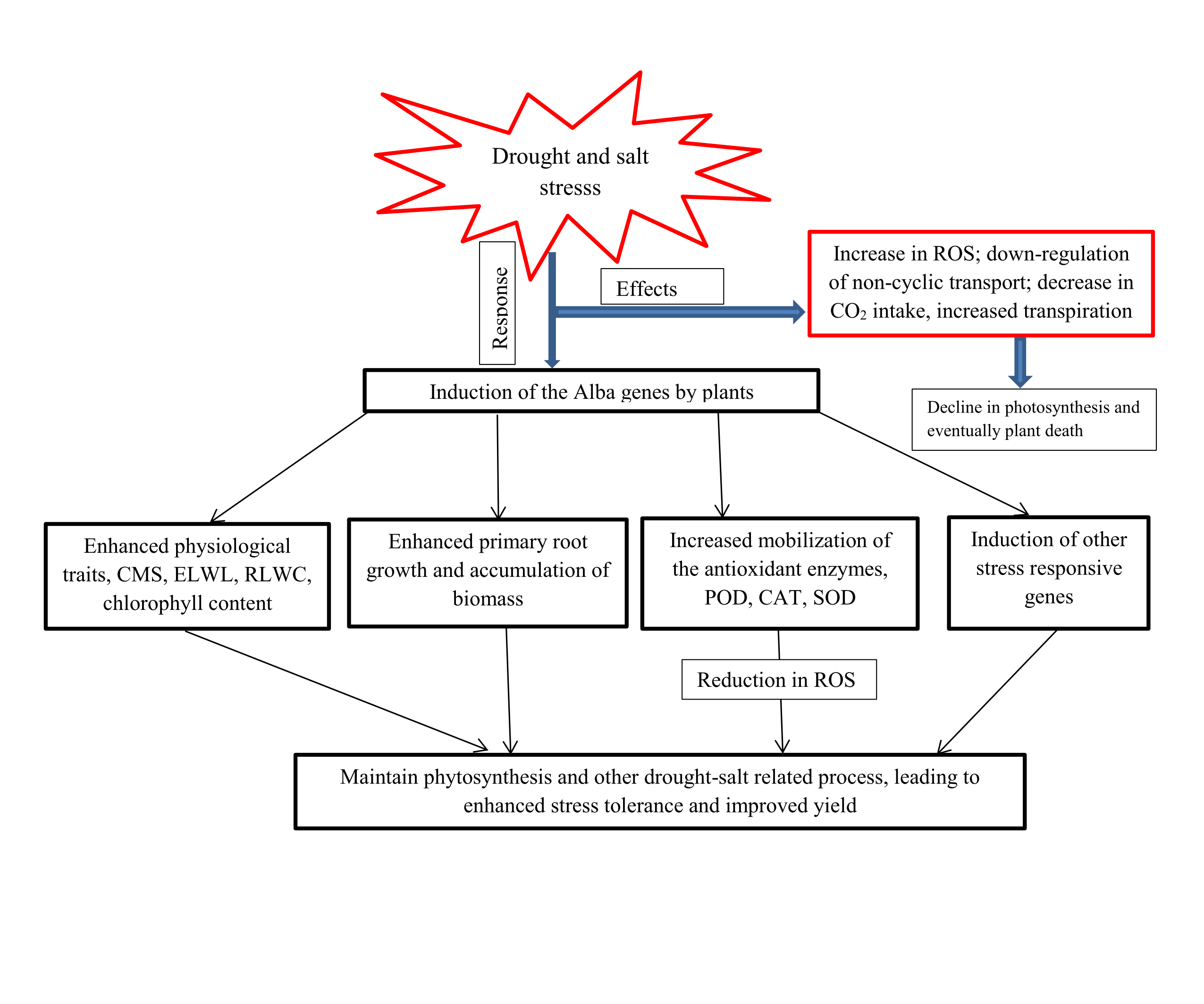

Supplement: Supplementary file 12 [file Image_6.tif]
