# Supplementary material for: Knockdown of ghAlba_4 and ghAlba_5 Proteins in Cotton Inhibits Root Growth and Increases Sensitivity to Drought and Salt Stresses
Source: Front Plant Sci. 2019 Oct 15;10:1292. doi: 10.3389/fpls.2019.01292 (PMC6804553; doi:10.3389/fpls.2019.01292)
Supplement: Table S1 — Protein sequences for the Alba proteins used in the phylogenetic tree analysis. Gh: G. hirsutum; Gorai: G. raimondii, Cotton_A: G. arboreum; Thecca: Theobroma cacao, AT: Arabidopsis thaliana, Sobic: S. bicolor, Glyma: G. max, Potri: P. trichorcarpa and LOC_Os: Oryza sativa. [file Table_1.docx]

**Table S1:** Protein sequences for the Alba proteins used in the phylogenetic tree analysis. Gh: *G. hirsutum*; Gorai: *G. raimondii*, Cotton_A: *G. arboreum*; Thecca: *Theobroma cacao*, AT: *Arabidopsis thaliana*, Sobic: *S. bicolor*, Glyma: *G. max*, Potri: *P. trichorcarpa* and LOC_Os: *Oryza sativa*

>Thecc1EG001307

MDRYQRVEKPKAETPINENEIRITTQGRMRNYITYATTLLQSNSNSMLSEANSGYSEKGSTEIVLKAMGRAINKTVMIAELIKRRIADLHQNTSIGSTDITDMWEPLEEGLLPLETTRHVSMITIALSKKELDTSSTGYQPPLPADQVKPLNEFEDDVEASPRTRGRGRSGRGRVRGRGNYGVGEYNGDGWDGGRGFGGRGRGRGRGRSFPGRGRGYGVGGYYGYGESDAPHVQGRGRGRGRGRGRGRGRYFRSDGPVQENAA*

>Thecc1EG002984

LANSLRFLFLVCLFCFKAFTLPLSLCCFVLLLGFNQGFLLLPFPSISTNKQPMDRYQKVEKPKPESPINENEIRITSQGAIRNYINYAIALLQDKRVKEIVLKAMGQAISKTVAIAEIIKVGFLFYHPCILFSHFPGLVICLVKQKRIPRLHQDTAISSVSITDVWEPIEEGLVPVEMTRHVSMISITLSARELNKNSAGYQAPHYVEQPKPQYHYQQQQPPKQARIPYNAVNEDSYGQGRGRGRGRGRNWARGGYGNYQDNGGYSNWGRGGGRGRGWGYRGSGYERGRGGGGRGYSRGRGRMGGGRSRGG

>Thecc1EG006429

MDRYQKVEKPKAETPINENELRITAQGRMRNYISYAMTLLQEKGANEIVLKATGRAINKTVMIAELIKRRIVGLHQNTSTGSIDITDTWEPLEEGLLPLETTRHVSIITITLSKKELDSSSIGYQPPIPADLVKPLAEFEDNEGETSPDIQGKGYGHSDQEKYGGNINGVVEHRNGGWDGGRGYGGRGRGRGRGRGFRGRGRGGYGGGNMQWDSGYYNGNGPSGPLPGQGRGRGRGRGRGRGRGQGFRSDGPFQKTA*

>Thecc1EG020377

MGQAISQTVAISEIIKKRIPGLHQETSISSMSITDMWELMEEGLVPYYLRLEMTRQVSLISISLSTKELNKSSPGYLAIKLHLMSSRTNISLSNSKTKCTFFLFQEKQVGEVVLKAMGQAISKMVAIAEIIKKRIPGLHQETSISSMSITDMWEPMEEGLVPLEMTCQVFLILISLSTKELNKSSPGYLGFPLHFLYGQIHCQHHTSMLRSNIVGKCFITRDQAPSHDQQNQYQSQQQQNQMHVPVTGLLKIHMDEDELMVEGEDRDGAGPVTGTIQRMVVIGIRGEEVDEAEVGAIVVGMEEAGVEGKAMVMDEDGAVAVDMVVDNRLNSGIVLM*

>Thecc1EG026310

MAVETVAAAEGDAQKKNRIQVSNTKKPLFFYVNLAKRYIQQHNEVELSALGMAITTVVTIAEILKNNGLAIEKKVMTSTVGMKDENKGRVVQKAKIEIVLGKSEKFDLLMNAANVPPEGASKDEE*

>Thecc1EG034281

MDRYQRVEKPKAETPIDENEIRITSQGRMRSYITYAMTLLQEKGSNEIVFKAMGRAINKTVTIVELIKRRIVGLHQITSIGSTDITDIWEPLEEGLLPLETTRHVSMITITLSKKELNTSSVGYQPPLPADQVKPSAEIDHEGEGSPNGRGRGRGGRGRPRSRGNGFVSVEYEDGGWDRNRGYARGRGRGRGRGFRGRGRGGYNGPQVDRQQDVGYNYEAPPQGGRGRGRGRGYRGRGRGFRSNGPIQAAA*

>Thecc1EG038055

MEEITQGVNNMNLGADSHKKNRIQVSNTKKPLFFYVNLAKRYMQQHNEVELSALGMAIATVVTIAEILKNNGLAVEKKIMTSTVDMKEDSRGRPVQKAKIEILLGKTENFDELMAASAEERDAVDGEEQS*

>Thecc1EG038396

MEGITEGVNNLNITDSSASNKKNRIQVSNTKKPLFFYVNLAKRYMQQHNEVELSALGMAIATVVTIAEILKNNGLAVEKKIMTSTVDMREESGGRPVQKAKIEILLGKSEKFDELMAASAEEVLDNEEQS*

>Glyma.01G234800

MEMTRVNAKSNNSNAKTNGVKEKAKKIVRIQVSKTKKPFFFYLNLAKKYIKQGNDVELSALGMAIPTVVIISEILKSNGWAFEKNITTSTVAAKEDKEGREIPKAKLGVLLGKAGDMDQSTVDASLGKNADD*

>Glyma.03G248100

MPTTVAVSVPQNQNTNHGVESPKKNKIQVSNTKKPLFFYVNLAKRYIQQRNEVVLSALGMGITTVVTIAEILKNNGLAIEKKVSTSSVTMKDETKGRLVQKAKIEIVLEKTEKFDSLTAVANTESKVVAADDDNHEKK*

>Glyma.04G217100

MDRYQRVEKPKAESPINENEIRVTSQGRTRNYITYATTLLQEKGSSEIVLKAMGRAINKTVMIAELIKRRIVGLHQNTQIGSTDITDTWEPLEEGLLPLETTRHVSMITITLSKKELDTSSTGYQPPLPVDQVKPLNEYDEEGEGSPRIRGRGRGRGRGRGRGFYNGGMEYGDGWDGGRGYGGRGRGRARGHAFRGRGRGRGYGVQPVGYYDYGEYDAPPPPRGRGRGRGRGRGRGRGRDQVPAA*

>Glyma.06G148800

MDRYQRVEKPKAETPINENEIRVTTQGRMRNYITYATTLLQEKGSSEIVLKAMGRAINKTVMIAELIKRRIVGLHQNTQIGSTDITDTWEPLEEGLLPLETTRHVSMITITLSKKELDTSSAGYQHPLPVDQVKPLNEYEEEGEGSPRMRGRGRGRGRGRGRGFYNGGMEYGDDWDGGRGYGGRGRGRARGRAFRGRGRGYGVQPVGYYDYGEYDAQPPPRGRGRGRGRGRGRGRDRGPPA*

>Glyma.07G031600

PQEGEKFQGGVNTINISDSYKKNCHSRSSNTKKPLFSTSISPKRYMQQHNEVELSALGMAIATVVTVAEILKNNELAVEKKITTSTVDIKDDSRGRPVQKAKIEILLGKTEKFDELMAAAAAAAAEDGENGDVEEHAA*

>Glyma.08G211200

MLLFSHSHTTAMEAITEGVNNINISDSYKKNRIQVSNTKKPLFFYVNLAKRYMQQHNEVELSALGMAIATVVTVAEILKNNELAIEKKITTSTVDIKDDSRGRPVQKAKIEILLGKTEKFDELMAAAAAAAEDGENGDVEEHTA*

>Glyma.08G356000

MDRYQKVEKPKPESPINENEIRITTQGAIRNYITYASSLLQEKHASEIVLKAMGQAISKTVAIAEILKLHQDTGISSVSIVDTWEPIEEGLVPVEMTRHVSMISITLSTRELDENSPGYQAPSNVEQPKQHSNYQQQSIKPARVPYNAVHEDSYGRGRGRGRGRGRGRGRNWGRGGYGYQGGYANYQGYGYYQGGYANHQDNGGYSNRGRGGGRGRGWGYRAAMVLLWQNGVVEFC*

>Glyma0G163500

MATVAAVPALTANDSHKKNRIQVSNTKKPLFFYVNLAKRYIQQHNEVELSALGMAIATVVTIAEILKNNGLATEKKVLTSTVGMKDENKGRLVQKAKIEIVLGKSDKFDNLMSPPAPTESEEAAADDDDKKE*

>Glyma3G364200

MEGITEGVNNININISDSYKKNRIQVSNTKKPLFFYVNLAKRYMQQHDEVELSALGMAIATVVTVAEILKNNGLAVEKKITTSTVDIKDDSRGRPVQKAKIEIVLGKTENFDELMAAAAAEDGENRDVEEQSA*

>Glyma4G221700

MDRYQKVEKPRAETPIDENEIRITSQGRMRNYITYAMSLLQEKGSNEIVFKAMGRAINKTVTIVELIKRRIVGLHQNTAIGSTDITDTWEPLEEGLLPLETTRHVSMITITLSKKELDTSSVGYQPPLPADQVKAATDFDYEGEGSPNGRVRGRGGRGRGRGRGNGFISADYEDGGWDRNRGNARGRGRGRGRGFRGRGRGGYNGPHVDMQDGGYNQDVPQGRGRGRGRGGYRGRGRGFRSNGPIQAAA*

>Glyma5G009400

MEDITEGVNNINISDSYKKNRIQVSNTKKPLFFYVNLAKRYMQQHDEVELSALGMAIATVVTVAEILKNNRLAVEKKIRTSTVDIKDDSRGRPVQKAKIEIVLGKTANFDELMAAAAAEDGENGDVEEQSA*

>Glyma7G260600

MDRYQKVEKPRAETPIDENEIRITSQGRMRNYITYAMSLLQEKGSNEIVFKAMGRAINKTVTIVELIKRRIVGLHQNTAIGSTDITDTWEPLEEGLLP*

>Glyma8G173100

MDRYQKVERPKPESPINENEIRITTQGAIRNYITYATSLLQEKHAREIVLKAMGQAISKTVAIAEILKKRIPKLHQDTGISSVSIVDTWEPIEEGLVPVE

MTRHVSMISITLSTRELNKNSPGYQAPSNVEQPKPHSNYQQQSIKPAQVHNAVNEDLYGRGRGRGRGRGRGRNWGRGGYGYQGGYGNYQGYGYYQGGYGNYQDNGGYSNRGRGGGRGRGWGYRGTGYEGGRGGGYEGGRGGGYEGGRGGGYEGGRGGGYEGGRGGGYEGGRGGGYEGGRGGGYEGGRGGGYERGRGGGRGYGRGRGRMGGRTRGGGGGNQA*

>Glyma9G246100

MLATVAASVPQNQNTNHEVESPKKNKIQVSNTKKPLFFYVNLAKRYIQQRDEVVLSALGMGITTVVTIAEILKNNGLATEKKISTSSVSLKDENKGRLVQKAKIEIVMEKTEKSTESEAAATDDGNEKK*

>Glyma.20G226100

MATVAAAPALTANDSHKKYRIQVSNTKKPLFFYVNLAKRYIQQHDEVELSALGMAIATVVTISEILKNNGLATEKKVLTSTVGMKDENKGRLVQKAKIEIVLGKSDKFDNLMSPPAPTESEAAAADDDHDKK*

>Potri.002G017600

MQRLLCMVEEEVREKRRLLYFSKSIFKGYYHRSCMGASNISILYWRSVIFSRASASGLGRKRRNEAVEEGSFLAEQASLCFYFSRKMDRYQRVEKPRADKTIDENEIRITSQGRMRSYISYAMTLLQEKGSNEIVFKAMGRAINKTVTIVELIKRRIVGLHQITSIGSTDITDTWEPLEEGLLPLETTRHVSMITINLSKKELNTSSAGYQPPLPAEQVKAFTEFEYEGDGSPRGRGRGRGGRGRARGRGNGFVSAEHEDGGWDRNRGYPRGRGRGRGRGFRGRGRGGFNGPHADTQQDGGHNYEGPPQGRGGYNYEAPPQGRGGYNYEAPPQGRDGYNYEAPPHGRGRGRGRGNRGRGRGFRSNGPIPAAA*

>Potri.002G066100

MDRYQRVEKPRNETPINENEIRITTQGRMRNYITYATTLFQEKGSDEISLKAMGRAINKTVMIAELIKRRIAGLHQNTSIGSTDITDTWEPLEEGLLPLETTRHVSVITVTLSKKELDTSSTGYQSPIPADQVKPLAEYDYEGEGGSPRRQGRGRGGRGIARGRGNNSNGVVEHNGDGDRVGGRGYGGRDGGRGYGDRDGGRGYGGRDGGYGYGGRDGGYGYGGRGRGRGRGRGYRGRGRGYGGGYMQQQSGGYNDYGGGAFVGQGSGRGRGRGRGRGRGRGFRLDGPAQPAE*

>Potri.002G240600

MDRYTRVEKPKPESPINENEIRITSGGPLRNYISYGTSLLQEKHVKEIVLKAMGQAISKTVSVAEGIKRRNPRLHQDTTISSVSITDVWEPLEEGLVPVEQTRQVSMITITLSFRELNKMSPGYQVPHSMKQPKQQDHSSSSSNLDKHVVPTMLFLKVIRMAEVIHMAEDVVEGEDGTGAGVDMDTETIRVTTTKEAIGEIITGTIRETIKIMVVTQIRAVVGVGAGIGVIVELDMKEAEVEGAEAMAKVVEGWPIAQGAAAAAEVAMATATATATKLKKRMVFSPFASAKCLPRPVGWILIPILVLMA*

>Potri.005G056000

MTMETVAVAPTPTPTPTTHQTMNNDTTTTLAQQKNNRIQVSNTKKPLFFYVNLAKRYMQQYNEVELSALGMAITTVVTIAEILKNNGLATEKKVLTSTVCMKDENKGRQVQKAKIEIVLGKSEKFDSLMNAANAAPEEEAAKEKDDEK*

>Potri.005G194600

MDRYQRVEKPRTETPINENEIRITTQGRMRNYITYATTLFLEKGSDGISLKAMGRAINKTVMIAELIKRRIAGLHQNTSIGSTDITDTWEPLEEGLLPLETTRHVSVITITLSKKELDTSSTGYQSPIPADQVKPLAEYDYEGEGGSPRRHGRGRGGRGMARGRGNTSNGVEYNGDGGWDGGRGYAGRGRGRGRGRGYRGRGRGYGGGYMPQQSGGYNDYGGGAFVGQGRGRGRGRGRGRGRGRGFRPDGPAQAAE*

>Potri.005G244300

MDRYQRVEKPRADTPIDENEIRITSQGRMRSYISYAMSLLQEKGSNEIVFKAMGRAINKTVTIVELIKRRIVGLHQITLIGSTDITDTWEPLEEGLLPLETTRHVSMITITLSMKELNTSSVGYQLPLLAEQVKAFTDFEYEGDGSPSGRGRGRGGRGRARSRGNGFVFAEHEDGGWDRNRGFPRGRGRGRGRGFRGRGRGGFNAPHGDTQQDGGYNYDAPPQGRGIFPRHFFLAVHDQYGYPSEFIFYHKKES*

>Potri.006G252000

MEVITEGVNNLNIAAAAATTDSANNKKNRIQVSNTKKPLFFYVNLAKRYMQQHNEVELSALGMAIATVVTIAEILKNNGLAVEKKIMTSTVDMREETGGRPVPKAKIEILLGKTEKFDELMAAAAAEEAAEAEEQN*

>Potri.013G042900

MTMETLAVAPTPTPTPTPTPTPTPTTETVNNDTAIALAQQKKNRIQVSNTKKPLFFYVNLAKRYMQQYNEVELSALGMAITTVVTIAEILKNNGLAIEKKVLTSTVGMKDENKGRQIQKAKIEIVLEKSEKFDSLMNAVSSAPEEEAAKDNKDDEKQ*

>Potri.018G029300

MEGITEGVTNLNITAAADSGNNKKNRIQVSNTKKPLFFYVNLAKRYMQQHNEVELSALGMAIATVVTIAEILKNNGLAVEKKITTSTVDMREETGGRPVPKAKVNHRIALCWTTFVRHAVRIYLLWHAADGILPNKDFTNFGIFSALLSLGNLGFLA*

>Sobic.001G099800

MDRYQRVERPRNESTIEENEIRITAQGLIRNYVSYATSLLQERRIKEIVLKAMGQAISKSVAVAEIIKKRIPGLHQDTNISSVSITDVWEPIEEGLVPLEMTRHVSMISITLSPRELDKQTPGYQAPVYVEQPRQQQGPPLQRQPRRPPGQQFQQLEYEDSYARGRGRGRGRGRGRGWGWGRGGYGGYGGYGNNQGGYNQGGGYYDNQGGYGGYDNQGGYGGGYGYNQGRYGNYQENGGYNRGRGGGMRGRGNWGYRGGYDGGRGGGYEGGRGGGYEGGRGGGYEGGRGGGYEGGRGGGYEGGRGGGYEGGRGGGYEGGRGGGAPGGRGYGGRGRGRMGGRGRGN*

>Sobic.001G493400

MDRYHRVEKPRPEAAAISENEIRITTQGLIRNYVTYATSLVQEKRVKEIVLKAMGQAISKTVAIAEIIKKRIPGLHQDTIISSVSITDVWEPIEEGLVPLEMTRHVSMISISLSPKELNKNSPGYQAPLHLDLKPQRYQQPQQYQQHQPRQNPIQTDSYGRGRGRGRGRGRGWGSRGGYGGGYGGYEYDNQGGYGGYGHQGGYGHQGGYGNQGGYGHNQDGYGGYGYNQGGYGGYENGGWNYNRNRGGGGGGGRGRGNWGYGGPGYDRGGRGAGGPGGRGYVRGRGRMGGGRGRGNQNY*

>Sobic.002G281600

MDRYQRVEKPREEAPIKENEIRITTQGRMRNYITYATALLQDKGSDEVVFKAMGRAINKTVMIAELIKRRIVGLHQNTTTGSTDITDMWEPLEEGLLPLETTRHVSMITITLSKKELDTSSIGYQSPLPADEVKPLVEYDNDEDAHSPGGRGRGRGGRGRGRGRGRGRGGRGNGYNDYADVGWEDDHAPAYMGNGYPRGRGRGFRGRGRRGGYNGQPDYQQDGGYYEEAPVHAPARGRGRGRGRGPSRGRGRGGNANGVMHAAAPGA*

>Sobic.003G049800

MVVEEITEGVKNLAVAGDAAAASGGEGQRRGGGGSSNRIQVSNTKKPLFFYVNLAKRYMQQHGDVELSALGMAIATVVTVAEILKNNGFAVEKKIRTSTVEINDESRGRPFQKAKIEIILGKSDKFDELMAAAAEERGEVEDGEEQA*

>Sobic.004G085200

MEEVTEAVNNLSISGGGATAGAGAGAEGHKKNRIQVSNTKKPLFFYVNLAKRYMQLHNEVELSALGMAIATVVTVAEILKNNGLAVEKKIMTSTVDVKDETRPRPIQKAKIEILLGKTDKFDELMAAAAEEREANEAEEQS*

>Sobic.005G050600

MDRYHRVEKPRNDTPISQNEIRITTQGRMRNYISYGMSLLEENGHDEINIKAMGRAINKTVMVVELIKRRVGGLHQNTATESVDITDTWEPLEEGLLPLETTRHVSMITVTLSKKPLDTSSPGYQPPIPAEEVKPAFDYDHEESYPTGRGRGRFGGRRGRGRGMSNGPPPPAYGYNDEWEEDGDYYNRGRGRGRSRGRGGRGRGGYYGGGRRGGYGYDYGYGGRGGYYEEQDEYYDEPEEYAPPPGRGRGRGRRGMPWRGRGGRGPPRGGRGGYY*

>Sobic.006G080000

MDRYHRVEKPREEEAPIGANEIRITAQGRPRNYITYALALLQDNATDDIVIKAMGRAINKTVVIVELLKRRIAGLHQNTSIESINITDTWEPLEEGLVTLETVRHVSLITIKLSKKELDTSSPGYQPPIPADQVRPAAEFDQDAEAVPSGRGRGRGRRGRGRGRGRGFSNGGVDYHDEFGEPEEAPRGYGGRGRGRGGRGSFAPGRGYGGDNYAMEEAGGYDDGYNAPPMQGYEGGRGRGRGRGRGRGRGRGSQGQGPPQQ*

>Sobic.008G099000

MQAVRPAAEGEEAQAQEQAVREEVAEVKREVAKAHEEEAAPEEKDVAVVGEEADAEAEAEAETEGEAEAEAEAGASAKKNRIQVSTNKKPLYFYVNLAKRYMQNYDEVELSALGMAIGTVVTVAEILKNNGLATEKKILTSTIGTKDESKGRLVRKAKIEILLCKSENFNSIMSSKKSDRPKSAEEEIKV*

>Sobic.009G063200

MSGGDMAAQAGGDAQQQQQQAVGGNRIQVSSSKKPLFFYVNLAKKYMQQHGDVELSALGLAISTVVTIAEILKNNGLAVEKKIRTSTVEIIDETKARPIQKAKIEIVLGKTDKFEELMAANVGDANAGDGEEQT*

>Sobic.010G184000

MEEVTEGVNNLAITEPHKKNRIQVSNTKKPLFFYVNLAKRYMQQHEEVELSALGMAIATVVTVAEILKNNGLAVEKKIMTSTVDVKDDTRARPIQKAKIEIVLGKTDKFDELMAAADAEREAAEAEEQS*

>Gorai.002G047400

MDRYQRVEKPKAETPIDEKEIRISSQGSMRNYISHALTLLQEKGSNQIVFKAMGKAINKAVAIVELIKKRIVGLHQITSIGSTDITDMWEPSEEGLVPLETTRHVSIIIITLSKIELNMSSAGYQPPLPANQVKAGSHNDRDGRRMPRSRGNAEYEDGGRNHNRGYDRGRGRGSRGRGRGRGGYNGQQADRMEDGGYNYEAPPQGGRGKGYRGRGRGFTSNRPIQAAA*

>Gorai.002G121600

MDRYQRVEKPKAETPIDENEIRITSQGRMRSYITYAMTLLQEKGSTQVVFKAMGRAINKTVTIVELIKRRIVGLHQITSIGSMDITDMWEPLEEGLLPLETTRHVSMITITLSKIELNTSSVGYQPPLPADQVKASTEVDHEGAEGSPNDRGRGRGGRGRPRSRGNGFVSAEYEDGSWDRTRGYARGRGRGRGRGVRGRGRGGYNGPQFDRPQDEGYNFEAPPQGGRGRGRGRGYRGRGRGFRSNGPIHAAA*

>Gorai.002G140000

MDQYQKVEKPKAETPINENDLRIIVQGRMRNYISYAMTLLQEKGAKKIVLKATSRAINKTVMIAELIMVYGALRMTVKIFLMWNSKMVIDGGEDTTVATSWLEASNLVAVPCIWLLLS*

>Gorai.002G206900

MDRYQKVEKPKAETPINENELRITAQGRMRNYISYAMTLLQEKGANEIVLKATGRAINKTVMIAELIKRRIAGLHQNTSTGSIDITDTWEPLEEGLLPLETTRHVSIITITLSKKVLDSSSIGYQPPIPTDQVKASAEIEGNEGEDSADTQGKGHVGQGKYGGNINGGMVDHRNGGWDGGRGYGGRGWGRGRGRGSRGRGRGYGGGNMQRDSGYYNGNDPSGPLPGQGRGGRGRGRGRGRGRGPPGQGFRSDGPFQKAA*

>Gorai.003G023400

MDQYQKVEKPKAETLINENELRIIVQGRMRNYISYAMTLLQEKGANKIVLKATGRAINKTVMIAELIMVYGALRMTVKIFLMWNSKMVIDGGEDTTVATSWLEASNLVAVPCVWLLLS*

>Gorai.004G058400

MDRYQKVEKPKSESPINDNEIRITSQGAIRNYINYAIALLQEKQAKEIVLKAMGQAISKTVAIAEIIKKRVPQLHQDTAISSLSITDVWDPIEEGLVPVEMTRHVSMISITLSTGELNKNSAGYQPPHFVEESKPQYHYHQQQSQKQARIPYNSVNEDSYGRARGRGRGRGRSWGRGGYGNYQDNGGYSNWGRGGGRGRGWGYCGSGYERGRRGGGRGYNRGRGRMHGGRSRGGGGGY*

>Gorai.004G274000

MEGITEGVNNLNIMDSSASNNNNKKNRIQVSNTKKPLFFYVNLAKRYMQQYNEVELSALGMAIATVVTIAEILKNNGLAIEKKIMTSTIDMREELGGRPVQKAKIEILLGKSEKFDELMAAAAAEDALENEEQS*

>Gorai.005G046900

MESTVAEEVSESPMAQEEVKNEATIIAGDDGNMENGKNPVLVAVVATAEVASIISPAKTVESPQEIKNMKNEKKKKQKKEVQVSNTKKPFIFYLNRAKRYINEFNEVELCGLGMAIPTVVTIAEILKRNGFAIQKGIMTSTVLSTQEDRKGRQIEKAKIEIVLGKAEKFGAMNAVVTPTKAAD*

>Gorai.007G063100

MDRYQRVEKPKAEIPIIENEIRITTQGRIRNYITYATTLLLEKGSNEIVLKAMGRAINKTVMIAELIKRRVADLHQITSIGSTDITDMWEPLEEGLLPLEITRHVSMITVTLSKKELDMSSTGYQPPLQADQVKPLNEYEDDGAPEPPPKARGRGRGGRGRIRAKGEYNGDGLGGKGRGRGRGRSFRGRGRGGAYGGGGYYGVYAESDATLTQVRGRGRERGRGGRGRGGGGRGRYSKTEPGPNQAKAA*

>Gorai.007G153000

MDRYQKVEKPKPESPINENEIRITSQGAIRNYINYAIALLQDKHVKEIVLKAMGQAISKTVAIAEILKKRIPRLHQDTSISSVSITDVWEPIEEGLVPVEMTRHVSMISITLSTRELNKNSVGYQAPHYAEQPKPQYHYQQQQLPKQGRIPYNAVNEDSYGRGRGRGRGRGRGRSWGRGGYGNYQDNGGYSNWGRGGGRGRGWGYRGAGYERGRGGGGRGFSRGRGRMGGGGRSRGGGY*

>Gorai.007G278700

MGVEAVAATGGGGGGGGGGGGGGVEAQKKNRIQVSNTKKPLFFYVNLAKRYIQQHNEVELSALGMAITTVVTIAEILKNNGLAIEKKVLTSTVGMKDENKGRVVLKAKIEIVLGKSEKFDLLMNASNVATETDPKDKE*

>Gorai.008G100300

MDRYQRVEKPKAEIPINENEIRLTTQGRMRNYITYATTLLQEKGSSEIVLKAMGRAINKTVMIAELIKRRIADLHQNTSIGSTDITDMWEPLEEGLLPLETTRHVSMITITLSKKELDVSSTGYQPPLSTDQVKPLNEFEEDGAPEATLGTRGRGRGARGRSIGRGIYGAVGGYNGDGWDGGRSVGSRARGRGRGNSFRGRGRGYGVGGYYDYGESDATLAQGRGLGRARGRRGRGRGRGLYSRSDLPVQANAA*

>Gorai.009G010100

MVGTTEGVDSSASNNKKNRIQVSNTQKPLFFYVNLAKRYMQQYNEVELSALGMAIATVVTIAEILKNNGLAVEKKIMTSTIDMREESGGRPVQKAKIEILLGKSEKFDELMAAAAEEALVDE*

>Gorai.009G018500

MEAITQGVNNINFGADSHKKNRIQVSHSKKPLFFYVNLAKRYMQQHNEVELSALGMAIATVVTIAEILKNSGLAVEKKITTATVDMKEESRGWPVQKAKIEILLGKTENFDELMVAAAEEREAVDGEYQS*

>Gorai.009G192000

MDRYQRVEKPKAVTPIDENEIRVTSQGRMRNYITYAMTLLQEMGSNQIVFKAMGRAISKTVTIVELLKKRIVGLHQITSIGSTDITDMWEPLEEGLLPLETTRHVPMISITLSKNELNTSSVGYQPPLPADQVKPSIKIDHKEGGSPNGRGRGRGGRVRSRSRGNAVVSAEYDDGGWDHNHGYASGRGRGRGRGSQGSGRGGYNGPQVGRLEDGGYNYEALPQGSRGRGRGRGYRGRGRGFRSNGPIQAAV*

>Gorai.010G064500

MEEITQGVNNINLVADSLKKNRIQVSNTKKPLFFYVNLAKRYMQQHNEVELSALGMAIATVVTIAEILKNNGLAVEKKITTSTVDMKEDSRGRPVQKAKIEILLGKTENFDELMAASAEEREIVDGEVQG*

>Gorai.012G160400

MDRYQKVEKPKADTPINENELRITAQGRMRNYISYAITLLQDKGANEIVLKATGRAINKTVMIAELIKRRIAGLHQNTSTGSIDITDTWEPLEEGLLPLETTRHVSIITITLSKKELDSSSIGYQPPIPADQVKPSAEFEDNEGGHINGGTVEHRNGGWDGGRGYGGRGRGRGRGRGRGFRGRGRGYGGGNMQRDSGYYNGNGPSGPLPAQGRGKPFAFNVFSYPVLVHSPFLSNHLLHQVVDEVEEGDVEAVVVRVSHPMVHSRKVLETARALILRFFCKTLALVNKKSCPFSTLIKALFFTWRICVVRDL*

>Gorai.013G105600

MEVITEGVNSLSIADSSPSNKKKNRIQVSNTKKPLFFYVNLAKRYMQQYNEVELSALGMAIATVVTIAEILKNNGLAVEKKIMTSTVDMREESGGRPVQKAKIEILLGKSEKFGELMAAAAAKDVLDNEEQS*

>Gorai.013G106200

MRNYISYAMTLLQEKGAKKIVLKATGRAINKTVMIAELIMVCGALRMTVKIFLMWNSKMVIDGCEDTTIATSWLEASNLVAVPCIWLLLS*

>Gorai.013G233800

MEEITQGVNNINLASDSHKKNRIQVSNTKKPLFFYVNLAKRYMQQYNEVELSALGMAIATVVTIAEILKNNGLAVEKKITTTTVDMKEDSRGRPVQKAKIEILLGKTENFDELMAAAAEERDGVVVEEEQQT*

>AT1G20220

MDKYQRVEKPKADTPIAENEIRITSMGRARNYITYAMALLQENKSNEVIFKAMGRAINKSVTIVELIKRRIPGLHQITSIGSTDITDTWEPTEEGLQTIETTRHVSMITITLSKEQLNTSSVGYQCPIPIEMVKPLAEIDYEGQDGSPRGRGGRRGRGGRGRGRGRGGRGNGPANVEYDDGGRGRGGRGNGYVNNEYDDGGRGRGGRGSGYVNNEYNDGGMEQDRSYGRGRGRGRGGGRGGRGRGGYNGPPPPYYEAQQDGGDYGYNNVAPPADHGYDGPPPQGRGRGRGRGGRGRGGGRGGFNRSNGAPIQAAA*

>AT1G29250

MEEITEGVNNMNLAVDTQKKNRIQVSNTKKPLFFYVNLAKRYMQQYTDVELSALGMAIATVVTVAEILKNNGFAVEKKIMTSTVDIKDDSRGRPVQKAKIEITLAKSEKFDELMAAANEEKEAAEAQEQN*

>AT1G76010

MDKYQRVVKPKADTPIDANEIRITSQGRARNYITYAMTLLQDKGSTEVVFKAMGRAINKTVTIVELIKRRIPDLHQNTSIGSTDITDTWEPTEEGLLPLETTRHVSMITITLSKIELNTSSVGYQCPIPIELVKPMGDIDYEGREGSPGGRGRGRGRGRGRGRGRGGRGNAYVNVEHEDGGWEREQSYGRGRGRGRGRSSRGRGRGGYNGPPNEYDAPQDGGYGYDAPHEHRGYDDRGGYDAPPQGRGGYDGPQGRGGYDGPQGRRGYDGPPQGRGGYDGPSQGRGGYDGPSQGRGGYDGPSQGRGGYDGPQGRGRGRGRGRGGRGRGGGRGGDGGFNNRSDGPPVQAAA*

>AT2G34160

MEEITDGVNNMNLATDSQKKNRIQVSNTKKPLFFYVNLAKRYMQQYNDVELSALGMAIATVVTVTEILKNNGFAVEKKIMTSTVDIKDDARGRPVQKAKIEITLVKSEKFDELMAAANEEKEDAEAQVQN*

>AT3G04620

MAMEVATPAPAPIPSERNIVLAPATTTTTATVETHKKNRIQVSNTKKPLFFYVNLAKRYIQQHNEVELSALGMAITTVVTISEILKNNGLATEKKVLTSTVGMKDETKGKMVQKAKIEIVLGKSDKFDSLVPPVTNGKTPEEEASAETEASVEAQEEVAAATEV*

>AT3G07030

MDRYQRVAKPKPESPINENEIRITSKGLIRNYISYATSLLQEKSVKDIVLKAMGQAISKTVAISEILKNKIPGLHQDIAISSISITDVWEPTEEGLFPVELTRHVSMISITLSLSELNKDSPGYQAPAQSDQSKPQYQPQQGRQARLPYNAYGEEGEVVAEGEAGEEVDMETTKGVMKEKTKGTIKKIIKTMKVGIQTRAEAVDVVDEAMAIVGGRGGYGGGRDGGYGGGRDDGYGERRNDGYGERRNDRYGGGRDDGYGGGRDDGYGGGRNDGYGGRRGGFRGGRGGGRDEGYGGGRGGYGGRSGGQGDGYGGGRGDGYGGGRGDGYGGGRGDGYGGGRVDRYDGGRRDGYGGGRYDGYGGGKSDGYGGGRGGYRGGRGGYGRGRGRMGNGGRSRDGASNQNEA*

>Gh_A01G0884

MDRYQRVEKPKAETPIDENEIRITSQGRMRSYITYAMTLLQEKGSSQVVFKAMGRAINKTVTIVELIKRRIVGLHQITSIGSMDITDMWEPLEEGLLPLETTRHVSMITITLSKKELNTSSVGYQPPLPADQVKASTEVDHEGEGSPNDRGRGRGGRGRPRSRGNGFVSAEYEDGSWDRTRGYARGRGRGRGRGVRGRGRGGYNGPQFDRLQDEGYNYEAPPQGGRGRGRGRGYRGRGRGFRSNGPIHAAA

>Gh_A01G1470

MDRYQKVEKPKAETPINENELRITAQGRMRNYISYAMTLLQEKGANEIVLKATGRAINKTVMIAELIKRRIAGFHQNTSTGSIDITDTWEPLEEGLLPLETTRHVSIITITLSKKVLDSSSIGYQPPIPTDQVKASAEIEGNEGEDSADTQGKGHGGQGKYGGNINGGMVDNRNGGWDGGRGYGGRAQGRGRGRGFRGRGRGYGGGNMQRDSGYYNGNDPSGPLPGQGRGGRGRGRGRGRGPPGQGFRSDGPFQKAA

>Gh_A02G0345

MESTVAEEVSESPMAQEEAKNEATIIAGDDGNMENGKNPVLVAVVTAAEVASLISPARTVESPQEIENMKNEKKKKQKKEVQVSNTKKPFIFYLNRAKRYINEFNEVELCGLGMAIPTVVTIAEILKRNGFAIQKGIMTSTVLSTQEDRKGRQIEKAKIEIVLGKAEKFGAMNAVVTPKKAAD

>Gh_A04G1077

MDRYQKVEKPKADTPINENELRITAQGRMRNYISYAITLLQEKGANEIVLKATGRAINKTVMIAELIKRRIAGLHQNTSTGSIDITDTWEPLEEGLLPLETTRHVSIITITLSKKELDSSSIGYQPPIPADQVKPSAEFEDNEGGNINGGMVEHRNGGWDGGRGYGGRGRGRGRGRGRGFRGHGRGYGGGNMQRDSGYYNGNGPSGPLPAQGRGEPFAFNVFLYPVLVHSPFLANHLLHQVVDEVEEGDVEAVVVRVSHPMVHSRKVLETTRALILRFFCRTLALVNKKSCPFSTLIKALFFTWRICVVRDL

>Gh_A05G0101

MEAITQGINNINMATDSHKKNRIQVSNSKKPLFFYVNLAKRYMQQHNEVELSALGMAIATVVTIAEILKNNGLAVEKKITTATVDMKEESRGRPVQKAKIEILLGKTENFDELMAAAAEEREAVEGEYQS

>Gh_A05G1575

MDRYQRVEKPKAVTPIDENEIRVTSQGRMRNYITYAMTLLQEMGSNQIIFKAMGRAISKTVTTVELLKKRIVGLHQITSIGSTDITDMWEPLEEGLLPMETTRHVSMITITLSKNELNPSSVGYQPPLPADQVKASIKIDHEEGGSPNGRGRGRGGRGRSRSRGNAVVSAEYDDGGWDRNHGYASGRGRGRGHGSQGSGRGGYNGPQVGRLEDGGHNYEALPQGSRRGRGRGYRGRGRGFRSNGPIQAAV

>Gh_A05G3960

MVGTTEGVDSSASNSKKNRIQVSNTQKPLFFYVNLAKRYMQQYNEVELSALGMAIATVVTIAEILKNNGLAVEKKIMTSTIDMREESGGRPVQKAKIEILLGKSEKFDELMAAAAEEALDDE

>Gh_A06G0483

MEEITQGVNNINLVADSHKKNRIQVSNTKKPLFFYVNLAKRYMQQHNEVELSALGMAIATVVTIAEILKNNGLAVEKKITTSTVDMKEDSRGRPVQKAKIEILLGKTENFDELMAASAEEREIVDGEVQG

>Gh_A08G0430

MDRYQKVEKPKSESPINDNEIRITSQGAIRNYINYAIALLQEKQAKEIVLKAMGQAISKTVAIAEIIKKRVPQLHQDTAISSLSITDVWNPIEDGLVPVEMTRHVSMLSITLSTGELNKNSAGYQAPHFVEESKPQYHYHQQQPQKQAQIPYNSVNEDSYGRGRGRGRGRGRSWGRGGYGNYPDNGGYSNWGRGGGRGRGWGYRGNGNERGRRGGGRGYNRGRGRMGGGRSRGGGGGY

>Gh_A08G2091

MEGITEGVNNLNIMDSSPSNNNNKKNRIQVSNTKKPLFFYVNLAKRYMQQYNEVELSALGMAIATVATIAEILKNNGLAVEKKIMTSTIDMREESGGRPVQKAKIEILLAKSEKFDELMAAAAAEDALENEEQS

>Gh_A11G0507

MDRYQRVEKPKAEIPIIENEIRITTQGRIRNYITYATTLLLEKGSNEIVLKAMGRAINKTVMIAELIKTRVADLHQITSIGSTDITDMWEPLEEGLLPLEITRHVSMITVTLSKKELDMSSTGYQPPLQADQVKPLNEYEDDGAPEPPPKTRGRGRGGRGRIRAKGEYNGDGLGGKGRGRGRGRSFRGRGRGGAYGGGGYYGVYSESDAALTQVRGRGRERGRGGRVRGGGGRGRYSKTEPGPNQAKAA

>Gh_A11G1257

MDRYQKVEKPKPESPINENEIRITSQGAIRNYINYAIALLQDKHVKEIVLKAMGQAISKTVAIAEILKKRIPRLHQDTSISSVSITDVWEPIEEGLVPVEMTRHVSMISITLSTRELNKNSVGYQAPHYAEQPKPQYHYQQQQPLKQARIPYNAVNEDSYGRGRGRGRGRGRGRSWGRGGYGNYQDNGGYSNWGRGGGRGRGWGYRGAGYERGRGGGGRGFSRGRERMGGGGRSRGGGY

>Gh_A11G2262

MGVEAVAATGGGGGGVEAQKKNRIQVSNTKKPLFFYVNLAKRYIQQHNEVELSALGMAITTVVTIAEILKNNGLAIEKKVLTSTVGMKDENEGRVVLKAKIEIVLGKSEKFDLLMNASNVATETDPKDKE

>Gh_A12G0762

MDRYQRVEKPKAEILINENEIRITTQGRMRNYITYATTLLQEKGSSEIVLKAMGRAINKTVMIAELIKRRIADLHQNTSIGSTDITDMWEPLEEGLLPLETTRHVSMITITLSKKELDLSSTGYQPPLSTDQVKPLNEFEEDGAPEAALGTRGRGRGARGRSRGRGIYGVVGGYNGDGWDSGRGVGGRARGRGRGNSFRGRGRGYGVGGYYDYGESDATLAQGRVLFNVEPFIGVVVVPGEEGGVGVGVGVVGIPGQTCQSKQTRLEKQKTL

>Gh_A13G1770

MEEITQGVNNINLAADSHKKNRIQVSNTKKPLFFYVNLAKRYMQQYNEVELSALGMAIATVVTIAEILKNNGLAVEKKITTTTVDMKEDSRGRPVQKAKIEILLGKTENFDELMAAAAEERDGVVVEEEQQT

>Gh_D01G0359

MDRYQRVEKPKAETPIDEKEIRISSQGSMRNYISHALTLLQEKGSNQIVFKAMGKAINKAVAIVELIKKRIVGLHQITSIGSTDITDMWEPLEEGLVPLETTRHVSIIIITLSKIELNMSSAGYQPPLPANQVKAGSHNDRDGRRMPRSRGNAEYEDGGRNHNRGYDRGRGRGSRGRGRGRGGYNGQQADRMEDGGYNYEAPPQGGRGKGYRGRGRGFTSNRPIQAAA

>Gh_D01G0922

MDRYQRVEKPKAETPIDENEIRITSQGRMRSYITYAMTLLQEKGSSQVVFKAMGRAINKTVTIVELIKRRIVGLHQITSIGSMDITDMWEPLEEGLLPLETTRHVSMITITLSKIELNTSSVGYQPPLPADQVKASTEVDHEGEGSPNDRGRGRGGRGRLRSRGNGFVSAEYEDGSWDRTRGYARGRGRGRGRGVRGRGRGGYNGPQFDRPQDEGYNFEAPPQGGRGRGRGRGYRGRGRGFRSNGPIHAAA

>Gh_D01G1707

MDRYQKVEKPKAETPINENELRITALGRMRNYISYAMTLLQEKGANEIVLKATGRAINKTVMIAELIKRRIAGLHQNTSTGSIDITDTWEPLEEGLLPHVSIIMITLSKKVLDSSSIGYQPPIPTDQVKASAEIEGNEGEDSADTQGKGHVGQGKYGGNINGGMVDHRNGGWDGGRGYGGRGQGCGRGRGSRGRGRGYGGGNMQRDSGYYNGNDPSGPLPGQGRGGRGRGRGRGRGRGPPGQGFRSDGPFQKAA

>Gh_D02G0408

MESTVAEEVSESPMAQEEVKNEATIIAGDDGNMENGKNPVLVAVVATAEVASIISPAKTVESPQEIKNMKNEKKKKQKKEVQVSNTKKPFIFYLNRAKRYINEFNEVELCGLGMAIPTVVTIAEILKRNGFAIQKGIMTSTVLSTQEDRKGRQIEKAKIEIVLGKAEKFGAMNAVVTPKKAAD

>Gh_D03G0593

MEVITEGVNSLNIADSSPSNNKKNRIQVSNTKKPLFFYVNLAKRYMQQYNGVELSALGMAIATVVTIAEILKNNGLAVEKKIMTSTVDMREESGGRPVQKAKIEILLGKSEKFGELMAAAAAKDVLDNEEQS

>Gh_D03G1718

EKGTKKIVLKATDRAINKTVMIAELIMVYGALRMTVKIFLIWNSKMVIDGCEDTTIATSWLE

>Gh_D04G2019

MDRYQKVEKPKADTPINENELRITAQGRMRNYISYAITLLQEKGANEIVLKATGRAINKTVMIAELIKRRIAGLHQNTSTGSIDITDTWEPLEEGLLPLETTRHVSIITITLSKKELDSSSIGYQPPIPADQVKPSAEFEDNEGGHINGGTVEHRNGGWDGGRGYGGRGRGRGRGRGRGFRGRGRGYGGGNMQRDSGYYNGNGPSGPLPAQGRGKPFAFNVFSYPVLVHSPFLSNHLLHQVVDEVEEGDVEAVVVRVSHPMVHSRKVLETTRALILRFFCKTLALVNKKSCPFSTLIKALFFTWRICVVRDL

>Gh_D05G0083

MVGTTEGVDSSASNNKKNRIQVSNTQKPLFFYVNLAKRYMQQYNEVELSALGMAIATVVTIAEILKNNGLAVEKKIMTSTIDMREESGGRPVQKAKIEILLGKSEKFDELMAAAAEEALDDE

>Gh_D05G1753

MDRYQRVEKPKAVTPIDENEIRVTSQGRMRNYITYAMTLLQEMGSNQIVFKAMGRAISKTVTMVELLKKRIVGLHQITSIGSTDITGMWEPLEEGLLPLETTRHVPMITITLSKNELNTSSVGYQPPLPADQVKASIKIDHKEGGSPNGRGRGRGGRVRSRSRGNAVVSAEYDDGGWDHNHGYASGRGRGRGRGSQGSGRGGYNGPQVGRLEDGGYNYEALPQGSRGRGRGRGYRGRGRGFRSNGPIQAAV

>Gh_D06G0537

MEEITQGVNNINLVADSHKKNRIQVSNTKKPLFFYVNLAKRYMQQHNEVELSALGMAIATVVTIAEILKNNGLAVEKKITTSTVDMKEDSRGRPVQKAKIEILLGKTENFDELMAASAEERELVDGEVQG

>Gh_D08G0518

MDRYQKVEKPKSESPINDNEIRITSQGAIRNYINYAIALLQEKQAKEIVLKAMGQAISKTVAIAEIIKKRVPQLHQDTAISSLSITDVWDTIEEGLVPVEMTRHVSMISITLSTGELNKNSAGYQPPHFVEESKPQYHYHQQQPQKQAQIPYNSVNEDNGGYSNWGRGGGRGRGWGYRGSGYERGRRGGGRGYNRGRGRMRGGRSRGGGGGGY

>Gh_D08G2460

MEGITEGVNNLNIMDSSASNNNNKKNRIQVSNTKKPLFFYVNLAKRYMQQYNEVELSALGMAIATVVTIAEILKNNGLAVEKKIMTSTIDMREESGGRPVQKAKIEILLGKSEKFDELMAAAAAEDALENEEQS

>Gh_D11G0585

MDRYQRVEKPKAEIPIIENEIRITTQGRIRNYITYATTLLLEKGSNEIVLKAMGRAINKTVMIAELIKRRVADLHQITSIGSTDITDMWEPLEEGLLPLEITRHVSMITVTLSKKELDMSSTGYQPPLQADQVKPLNEYEDDGAPEPPPKTRGRGRGGRGRIRAKGEYNGDGLGGKGRGRGRGRSFRGRGRGGAYGGGGYYGVYAESDATLTQVRGRGRERGRGGRGRGGGGRGRYSKTEPGLNQAKAA

>Gh_D11G1406

MDRYQKVEKPKPESPINENEIRITSQGAIRNYINYAIALLQDKHVKEIVLKAMGQAISKTVAIAEILKKRIPRLHQDTSISSVSITDVWEPIEEGLVPVEMTRHVSMISITLSTRELNKNSVGYQAPHYAEQPKPQYHYQQQQLPKQGRIPYNAVNEDSYGRGRGRGRGRGRGWSWGRGGYGNYQDNGGYSNWGRGGGRGRGWGYRGAGYERGRGGGGRGFSRGRGRMGGGGRSRGGGY

>Gh_D11G2569

MGVEAVAATGGGGGGGGVEAQKKNRIQVSNTKKPLFFYVNLAKRYIQQHNEVELSALGMAITTVVTIAEILKNNGLAIEKKVLTSTVGMKDENKGRVVLKAKIEIVLGKSEKFDLLMNASNVATETDPKDKE

>Gh_D12G0886

MDRYQRVEKPKAEIPINENEIRLTTQGRMRNYITYATTILQEKGSSEIVLKAMGRAINKTVMIAELIKRRIADLHQNTSIGSTDITDMWEPLEEGLLLLETTRHVSMITITLSKKELDVSSTGYQPPLSTDQVKPLNEFEEDGAPEATLGTRGRGRGARGRSIGRGIYGAVGGYNGDGWEGGRSVGSRARGRGRGNSFRGRGRGYGVGGYYDYGESDATLAQGCGLGRARGRRGRGRGLYSRSDLPVQANAA

>Gh_D13G2120

MEEITQGVNNINLAADSHKKNRIQVSNTKKPLFFYVNLAKRYMQQYNEVELSALGMAIATVVTIAEILKNNGLAVEKKITTTTVDMKEDSRGRPVQKAKIEILLGKTENFDELMAAAAEERDGVVVEEEQQT

>Gh_Sca129121G01

MDRYQKVEKPKADTPINENELRITAQGRMRNYISYAITLLQEKGANEIVLKATGRAINKTVMIAELIK

>Cotton_A_02961

MDRYQKVEKPKPESPINENEIRITSQGAIRNYINYAIALLQDKHVKEIVLKAMGQAISKTVAIAEILKKRIPRLHQDTSISSVSITDVWEPIEEGLVPVEMTRHVSMISITLSTRELNKNSVGYQAPHYAEQPKPQYQYQQQQPPKQARIPYNAVNEDSYGRGRGRGRGRGRGRSWGRGGYGNYQGAGYERGRGGGGRGFSRGRGRMGGGGRSRGGGY

>Cotton_A_03076

MEEITQGVNNINLAADSHKKNRIQVSNTKKPLFFYVNLAKRYMQQYNEVELSALGMAIATVVTIAEILKNNGLAVEKKITTTTVDMKEDSRGRPVQKAKIEILLGKTENFDELMAAAAEERDGVVVEEEQQT

>Cotton_A_03232

MDRYQKVEKPKSESPINDNEIRITSQGAIRNYINYAIALLQEKQAKEIVLKAMGQAISKTVAIAEIIKKRVPQLHQDTAISSLSITDVWNPIEEGLVPVEMTRHVSMLSITLSTGELNKNSAGCIEGHTAVLILEYVVDTSIRHGYQAPHFVEESKPQYHYHQQQPQKQAQISYNSVNEDSYGRGRGRGRGRGRSWGRGGYGNYPDNGGYSNWGRGGGRGRGWGYRGSGNERGRRGGGRGYNRGRGRMGGGRSRGGGGGY

>Cotton_A_08372

MESTVAEEVSESPMAQEEAKNEATIIAGDDGNMENGKNPVLVAVVAAAEVASIISPARTVESPQEIETMKNEKKKKQKKEVQVSNTKKPFIFYLNRAKRYINEFNEVELCGLGMAIPTVVTIAEILKRNGFAIQKDCLAMKTGIMTSTVLSTQEDRKGRQIEKAKIEIVLGKAEKFGAMNAVVTPKKAAD

>Cotton_A_08836

MDRYQKVEKPKADTPINENELRITAQGRMRNYISYAITLLQEKGANEIVLKATGRAINKTVMIAELIKRRIAGLHQNTSTGSIDITDTWEPLEEGLLPLETTRHVSIITITLSKKELDSSSIGYQPPIPADQVKPSAEFEDNEGGNINGGMVEHRNGGWDGGRGYGGRGRGRGRGRGRGFRGRGRGYGGGNMQRDSGYYNGNGPSGPLPAQGRGRGRGRGRGRGGGRGQGFTPDGPFQKGA

>Cotton_A_10898

MDRYQRVEKPKAEIPIIENEIRITTQGRIRNYITYATTLLLEKGSNEIVLKAMGRAINKTVMIAELIKRRVADLHQITSIGSTDITDMWEPLEEGLLPLEITRHVSMITVTLSKKELDMSSTGYQPPLQADQVKPLNEYEDDGAPEPPPKTRGRGRGGRGRIRAKGDTQGEYNGDGLGGKGRGRGRGRSFRGRGRGGAYGGGGYYGVYSESDAALTQVRGRGRERGRGGRGRGGGGRGRYSKTEPGPNQAKAA

>Cotton_A_11444

MEAITQGVNNINMATDSHKKNRIQVSNSKKPLFFYVNLAKRYMQQHNEVELSALGMAIATVVTIAEILKNNGLAVEKKITTATVDMKEESRGRPVQKAKIEILLGKTENFDELMAAAAEEREAVD

>Cotton_A_14221

MDRYQRVEKPKAETPIDEKEIRISNQGSMRNYISYALTLLQENGSNQIVFKAMGKAINKAVAIVELIKKRIVGLHQITSIGSTDIIDMWELLEGLVPLETTRHVKAGSHNDRDGRRMPRSRRSAEYDDGGRNHNRGYDRGRGRGSRGCRRGGCNGQQADRIEDGGYNYEAPPHGGRGKGYHGRGHRVTSNRPIQAAA

>Cotton_A_16250

MVGTTEGVDSSASNSKKNRIQVSNTQKPLFFYVNLAKRYMQQYNEVELSALGMAIATVVTIAEILKNNGLAVEKKIMTSTIDMREESGGRPVQKAKIEILLGKSEKFDELMAAAAEEALDDE

>Cotton_A_16451

MDRYQRVEKPKAVTPIDENEIRVTSQGRMRNYITYAMTLLQEMGSNQIIFKAMGRAISKTVTTVELLKKRIVGLHQITSIGSTDITDMWEPLEEGLLPMETTRHVSMITITLSKNELNPSSVGYQPPLPADQVKASIKIDHEEGGSPNGRGRGRGGRGRSRSRGNAVVSAEYDDGGWDRNHGYASGRGRGRGHGSQGSGRGGYNGPQVGRLEDGGHNYEALPQGRGRGRGYRGRGRGFRSNGPIQAAV

>Cotton_A_17439

MEEITQGVNNINLVADSHKNRIQVSNTKKPLFFYVNLAKRYMQQHNEVELSALGMAIATVVTIAEILKNNGLAVEKKITTSTVDMKEDSRGRPVQKAKPRGEINMEEALGLESRNPSAGGFIRDNQRNWVVDFGRNIGESLDSREFGGRHLEHI

>Cotton_A_20171

MEGITEGVNNLNIMDSSPSNNNNKKNRIQVSNTKKPLFFYVNLAKRYMQQYNEVELSALGMAIATVVTIAEILKNNGLAVEKKIMTSTIDMREESGGRPVQKAKIEILLAKSEKFDELMAAAAAEDALENEEQS

>Cotton_A_28567

MDRYQRVEKPKAETPIDENEIRITSQGRMRSYITYAMTLLQEKGSSQVVFKAMGRAINKTVTIVELIKRRIVGLHQITSIGSMDITDMWEPLEEGLLPLETTRHVSMITITLSKKELNTSSVGYQPPLPADQVKASTEVDHEGEGSPNDRGRGRGGRGRPRSRGNGFVSAEYEDGSWDRTRGYARGRGRGRGRGVRGRGRGGYNGPQFDRLQDEGYNYEAPPQGGRGRGRGRGYRGRGRGFRSNGPIHAAA

>Cotton_A_30556

MGVEAVAATGGGGGGVEAQKKNRIQVSNTKKPLFFYVNLAKRYIQQHNEVELSALGMAITTVVTIAEILKNNGLAIEKKVLTSTVGMKDENKGRVVLKAKIEIVLGKSEKFDLLMNASNVATETDPKDKE

>Cotton_A_33889

MDRYQKVEKPKAETPINENELRITAQGRMRNYISYAMTLLQEKGANEIVLKATGRAINKTVMIAELIKRRIAGLHQNTSTGSIDITDTWEPLEEGLLPLETTRHVSIITITLSKKVLDSSSIGYQPPIPTDQVKASAEIEGNEGEDSADTQGKGHGGQGKYGGNINGGMVDNRNGGWDGGRGYGGRARGRGRGRGFRGRGRGYGGGNMQRDSGYYNGNDPSGPLPGQGRGGRGRGRGRGRGPPGQGFRSDGPFQKAA

>Cotton_A_36717

MEGITEGVNSLNIADSSPSNKKNRIQVSNTKKPLFFYVNLAKRYMQQYNEVELSALGMAIATVVTIAEILKNNGLAVEKKIMTSTVDMREESGGRPVQKAKIEILLGKSEKFDELMAAAAAKDVLDNEEQS

>Cotton_A_40133

MDRYQRVEKPKAEILINENEIRITTQGRMRNYITYATTLLQEKGSSEIVLKAMGRAINKTVMIAELIKRRIADLHQNTSIGSTDITDMWEPLEEGLLPLETTCHVSMITITLSKKELDLSSTGYQPPLSTDQVKPLNEFEEDGALEAALGTRGRGQGARGRSRGRGIYGVVGGYNGDGWDSGRGVGGRARGRGRGNSFRGRGRGYGVGGYYDYGESDATLAQGRGKFEIRNFLLLSMVLFNVEPFIGVNYLPFCLKSCFPTFFSGRGWFIINVLKGWMPNFIMI

>LOC_Os01g07810

MAVEEITEGVRNLAVEGEPAAAAAAAGGGGEGAQRRAAGSSSNRIQVSNTKKPLFFYVNLAKRYMQQHGDVELSALGMAIATVVTVAEILKNNGFAVEKKIRTSTVEINDESRVRPLQKAKIEIVLEKSEKFDELMAAAAEEREAAEAEEQA*

>LOC_Os02g10810

MEEVTEAVGNLTIAAGEAAGAGGGAAEAHKKNRIQVSNTKKPLFFYVNLAKRYMQLHNEVELSALGMAIATVVTVAEILKNNGLAVEKKIMTSTVDVKDDSRSRPMQKAKIEIVLGKTDKFDELMAAAAEEREAAAAEAEAEEQS*

>LOC_Os03g06980

MDRYQRVEKPRPEAAVISENEIRITTQGLIRNYVTYATSLLQEKRVKEIVLKAMGQAISKTVAIAEIIKKRIPGLHQDTSISSVSITDVWEPIEEGLVPLEMTRHVSMISISLSPKELNKSSAGYQAPLHAEPIKPQRYQQTQQYQQQQHQSRPSQVQTDSYGRGRGRGRGRGRGWGGRGGYGGGYGGYDNNQGGYGGYGHQGGYGHQGGYGNQGGYGHNQGGYGGYGYNQGGYGGYENGGWNYNRNRGGGGGGGRGRGNWGYGGPGGYERSGPAYERGGRGGGSPGGRGYARGRGRMGGGRGRGNQNY*

>LOC_Os03g52490

MDRYQRVERPRPESAIEENEIRITAQGLIRNYVSYATSLLQDRRIKEIVLKAMGQAISKSVAVAEIIKKRVPGLYQDTNISSVSITDVWEPIEEGLVPLEMTRHVSMISITLSPRDLDKNSPGYQTPVYVEQPRQQPRLQQAPPPPQRQVRQPPPDYEDSYVRGRGRGRGRGRGRGWGRGGYGGYGGYGNNQGGYNQGGGYYDNQGGYGGYDNQGGYGGYDNQGGYGGGGYGYNQGRYGNYQENGGYNRGRGGMRGRGNWNYRGGYERGRGGGFPGGRGYGGRGRGRMGGRGGRGN*

>LOC_Os04g34940

MDRYQRVEKPREEAAIGANEIRITAQGRTRNYITYALALLQDNATDEIVIKAMGRAINKTVAIVELLKRRIVGLHQNTSIESIDITDTWEPLEEGLNTLETTRHVSLITITLSKKELDTSSPGYQPPIPADQVRPPTDFDQEAEAVPSGRGRGRGRRGRGRGRGFNNEDYDDEHGDAEVPQPQGYRGRGRGRGRRGSFGPGRGYGGDGFAMEEAGGYDDGEPNAPPMQGHEGGRGRGRGRGRGCGRGRGGGRGRGPPPPPQE*

>LOC_Os06g40040

MEEVTEAVSNLSITEPHKKNRIQVSNTKKPLFFYVNLAKRYMQQHNEVELSALGMAIATVVTVAEILKNNGLAVEKKIMTSTVDVKDDSRSRPMQKAKIEILLGKTEKFDELMAAAAEEREAAAAAEGEEQG*

>LOC_Os09g37006

MDRYQRVEKPREEAPIKENEIRITTQGRMRNYITYATTLLQDKGSDEVVFKAMGRAINKTVMIAELIKRRIVGLHQNTTTGSTDITDMWEPLEEGLLPLETTRHVSMITITLSKKELDTSSIGYQSPLPADKVKPLVEYENEEDAPSPAGRGRGRGGQGRGRGRGRGTRGNGYMDYADGGWEDDHAPPAYAGNGYTRGRGRGFRGRGRRGGGYGAQPDYQQDGGYYDEAPVHAPPRAGRGRGRGRGRGPVRGRGRGGNVNGVMHATAVGA*

>LOC_Os11g06760

MDRYQRVEKPRNETPISENEIRITAQGRMRNYISYGMSLLEENGHDEIVIKAMGRAINKTVMVVELIKRRIGGLHQITSTESIDITDTWEPLEEGLLPLETTRHVSMIAITLSKKALDTLSPGYQPPIPAEEVRPAFDYEHEESFPTNRGRGRGGGRRGRGRAMSNGPPAYDYGEEWEEEGDYYNYRGRGRGRFRGRGRGRGRGGYYGGGRRGGYGYDYGYGGRGDYYEDQGEYFEEPEDYPPPGRGRGRGRRGGGPGPFRGRGRGRGRF*

>LOC_Os12g30550

MQAVREEEEQVVEEVVRAGAVAEEEEGPEEKEVAMVGEEMAEAEHDEEEAEAGASAKKNRIQVSTNKKPLYFYVNLAKRYMQNYDEVELSALGMAIGTVVTVAEILKNNGLATEKKILTSTIGTKDESKGRLVRKAKIEILLCKSENFNSIMSSKKSDRPKSAEEEIKV*
