# Supplementary material for: Knockdown of ghAlba_4 and ghAlba_5 Proteins in Cotton Inhibits Root Growth and Increases Sensitivity to Drought and Salt Stresses
Source: Front Plant Sci. 2019 Oct 15;10:1292. doi: 10.3389/fpls.2019.01292 (PMC6804553; doi:10.3389/fpls.2019.01292)
Supplement: Table S2 — Details of the primers used for RT-qPCR analysis. [file Table_2.docx]

Table S2: Primer details as used for RT-qPCR analysis of the various cotton *Alba* genes under drought and salt stress conditions

| **Cotton species** | **Gene ID** | **Forward(5'-3')** | **Reverse(5'-3')** |
| --- | --- | --- | --- |
| *G. hirsutum (AD)* | Gh_A01G0884 | CAGAGGGTGGAGAAGCCGAAA | AGCAGAGTCATAGCATAAGTG |
|  | Gh_A01G1470 | AAACCACTCGCCATGTTTC | CTCTCCTTCATTTCCCTCA |
|  | Gh_A04G1077 | GGTGGAAATATGCAACGGGAC | AACACATTGAAAGCAAAGGGC |
|  | Gh_A05G0101 | AATGGCTATTGCGACGGTGGT | CTTCATGTCAACTGTTGCGGT |
|  | Gh_A05G1575 | GTGAAAGCATCCATAAAAATT | TACTCAGCAGAGACAACAGCA |
|  | Gh_A08G2091 | ATGGAGGGCATAACAGAGGGAG | AGGGGTTTTTTAGTATTGGAGA |
|  | Gh_A11G1257 | CAACTATGCTATTGCTCTTCTT | ACTGTCTTACTGATTGCCTGTC |
|  | Gh_A12G0762 | ATTAGGATAACAACACAAG | TAAGAACAATCTCACTGGA |
|  | Gh_A13G1770 | CCGCCGATTCCCACAAGA | GGGCAGACAACTCCACCT |
|  | Gh_D01G0922 | CTACAATTTTGAAGCCCCTC | GCCCATTAGATCTAAACCCA |
|  | Gh_D03G0593 | CCTCCAACAACAAGAAGAACC | AACAACTGTAGCAATAGCCAT |
|  | Gh_D04G2019 | AACTGACACATGGGAACCGCT | CTGATATCCTATTGAAGACGA |
|  | Gh_D05G0083 | CCTCCAACAACAAGAAGAACC | AACAACAGTAGCAATAGCCAT |
|  | Gh_D05G1753 | CATCTGTTGGGTATCAGCC | GCATTCCCTCTACTCCTTG |
|  | Gh_D06G0537 | TCGCCGATTCCCACAAGA | GGGCAGACAACTCCACCT |
|  | Gh_D08G0518 | GGGACAAGCAATCAGTAAG | GTCATCTCCACAGGAACAA |
|  | Gh_D08G2460 | GGAGGGCATAACAGAGGGAG | AGGGGTTTTTTAGTATTGGA |
|  | Gh_D11G1406 | ACTATGCTATTGCTCTTCTT | TGTCTTACTGATTGCCTGTC |
|  | Gh_D12G0886 | ATTAGGCTAACAACACAAG | TAAGAACAATCTCACTGGA |
|  | Gh_D13G2120 | ATTCAAGTCTCCAATACCAAA | GTCGCAATAGCCATGCCAAGG |
| *G. raimondii (D)* | Gorai.002G121600 | CACTTATGCCATGACTCT | GATGTAAACCAACGATTC |
|  | Gorai.002G206900 | ACCAGAAGGTGGAGAAGC | AAGCAAAGTCATGGCGTA |
|  | Gorai.004G058400 | GTATCAACCTCCACATTT | TCTCCCTCTACCACGACC |
|  | Gorai.004G274000 | GGAGGGCATAACAGAGGGAGT | GGGGTTTTTTAGTATTGGAGA |
|  | Gorai.007G153000 | CAACTATGCTATTGCTCTTCT | CTGTCTTACTGATTGCCTGTC |
|  | Gorai.008G100300 | ATCAGAGGGTGGAGAAGC | GAGGAGAGTGGTGGCATA |
|  | Gorai.009G010100 | CAACAACAAGAAGAACCGTA | CCATTCCAAGAGCAGAGAGT |
|  | Gorai.009G018500 | GGAGTAAACAACATCAAC | ATATACCTCTTAGCGAGA |
|  | Gorai.009G192000 | GCCGAAAGCAGTGACACCCAT | CGAGCCCATCTCCTGAAGCAA |
|  | Gorai.010G064500 | AGAAGAAGATCACAACCT | GCCATTAATTCATCAAAG |
|  | Gorai.012G160400 | TGGAAATATGCAACGGGACT | ACACATTGAAAGCAAAGGGC |
|  | Gorai.013G105600 | TCCAACAAAAAGAAGAACC | CAACTGTAGCAATAGCCAT |
|  | Gorai.013G233800 | CTCCAATACGAAAAAGCC | GCAGATAACTCCACCTCA |
| *G. arboreum (A)* | Cotton_A_02961 | TATGCTATTGCTCTTCTTC | AATGGCTACTGTCTTACTG |
|  | Cotton_A_03076 | CCGCCGATTCCCACAAGA | GGGCAGACAACTCCACCT |
|  | Cotton_A_08836 | ACCAAAAGGTAGAGAAGC | AAGCAGAGTGATGGCGTA |
|  | Cotton_A_11444 | TGATTCCCACAAGAAGAACAGG | CGTCGCAATAGCCATTCCAAGT |
|  | Cotton_A_17439 | GGTCGCCGATTCCCACAAGA | GGCAGACAACTCCACCTCAT |
|  | Cotton_A_20171 | AAACCCCTCTTTTTCTACG | TTTTCTCAACAGCCAGTCC |
|  | Cotton_A_28567 | GAGGGTGGAGAAGCCGAAA | CTGGAGCAGAGTCATAGCA |
|  | Cotton_A_33889 | ACCACTCGCCATGTTTCG | CCTCTCCTTCATTTCCCT |
|  | Cotton_A_36717 | GCCCTCCAACAAGAAGAACC | AACAACTGTAGCAATAGCCA |
|  | Cotton_A_40133 | AGGTATCAGAGGGTGGAGAA | TGGAGGAGAGTGGTGGCGTA |
