# Supplementary material for: Knockdown of ghAlba_4 and ghAlba_5 Proteins in Cotton Inhibits Root Growth and Increases Sensitivity to Drought and Salt Stresses
Source: Front Plant Sci. 2019 Oct 15;10:1292. doi: 10.3389/fpls.2019.01292 (PMC6804553; doi:10.3389/fpls.2019.01292)
Supplement: Table S3 — The predicted miRNAs found to target various cotton Alba genes cis-Alba. [file Table_3.docx]

Table S3: The predicted miRNAs found to target various cotton *Alba* genes

| **Cotton genome** | **miRNA** | **Target** | **miRNA start** | **miRNAend** | **Target start** | **Target end** | **miRNA_aligned_fragment** | **Target_aligned_fragment** | **Inhibition** |
| --- | --- | --- | --- | --- | --- | --- | --- | --- | --- |
| AD | ghr-miR3476-3p | Gh_A08G0430 | 1 | 21 | 692 | 712 | AGCCAACAACAUCAGUUCUAA | CAAGGGGUGGUGGUGGUGGCU | Translation |
|  | ghr-miR390a | Gh_D05G1753 | 1 | 21 | 496 | 516 | AAGCUCAGGAGGGAUAGCGCC | GCUGUUGUCUCUGCUGAGUAU | Translation |
|  | ghr-miR390b | Gh_A05G1575 | 1 | 21 | 496 | 516 | AAGCUCAGGAGGGAUAGCGCC | GCUGUUGUCUCUGCUGAGUAU | Translation |
|  | ghr-miR390c | Gh_D05G1753 | 1 | 21 | 496 | 516 | AAGCUCAGGAGGGAUAGCGCC | GCUGUUGUCUCUGCUGAGUAU | Translation |
|  | ghr-miR394a | Gh_D01G0359 | 1 | 20 | 442 | 461 | UUGGCAUUCUGUCCACCUCC | AGGAGUAGAGGGAAUGCUGA | Translation |
|  | ghr-miR394b | Gh_D01G0359 | 1 | 20 | 442 | 461 | UUGGCAUUCUGUCCACCUCC | AGGAGUAGAGGGAAUGCUGA | Translation |
|  | ghr-miR396a | Gh_D01G0922 | 1 | 21 | 465 | 485 | UUCCACAGCUUUCUUGAACUG | UGGUAGAGGAAGGCUGAGGAG | Cleavage |
|  | ghr-miR396b | Gh_D01G0922 | 1 | 21 | 465 | 485 | UUCCACAGCUUUCUUGAACUG | UGGUAGAGGAAGGCUGAGGAG | Cleavage |
|  | ghr-miR7484a | Gh_D04G2019 | 1 | 24 | 840 | 864 | UUUGUAUAUUAGA-UCAAAGAGCAA | CAAAACUUUGGCUUUAGUAAACAAA | Cleavage |
|  | ghr-miR7484b | Gh_D04G2019 | 1 | 24 | 840 | 864 | UUUGUAUAUUAGA-UCAAAGAGCAA | CAAAACUUUGGCUUUAGUAAACAAA | Cleavage |
|  | ghr-miR7486a | Gh_A12G0762 | 1 | 24 | 570 | 593 | AAGGAAGCGCUUUGUCCACGUGGA | AGGACGUGGAAGAGGUAAUUCCUU | Cleavage |
|  | ghr-miR7486b | Gh_D12G0886 | 1 | 24 | 570 | 593 | AAGGAAGCGCUUUGUCCACGUGGA | AGGACGUGGAAGAGGUAAUUCCUU | Cleavage |
|  | ghr-miR7498 | Gh_D11G2569 | 1 | 24 | 178 | 200 | AUGGUGACACAUGGUAGUCUCACA | GCAAUCACUACUGU-UGUCACUAU | Translation |
|  | ghr-miR7499 | Gh_D04G2019 | 1 | 24 | 50 | 73 | AUAUAAUUUUCGGUUAAUUCGGUU | ACGAGAAUGAACUGAGGAUUACAG | Cleavage |
|  | ghr-miR7502 | Gh_D02G0408 | 1 | 24 | 153 | 176 | UUUUUAACAGUAGAAAUGAAUGAA | UUCCAUCAUUUCUCCUGCGAAAAC | Translation |
|  | ghr-miR7504a | Gh_A01G1470 | 1 | 24 | 82 | 104 | UAUGAAACUGUGAUUCCACGUCAU | AGAAUGAGGAAUUACA-UUUCUUA | Cleavage |
| D | gra-miR164a | Gorai.002G121600 | 1 | 21 | 294 | 315 | CAUGUGCCUUG-GCUCUCCAUC | AUUGGAGACCACCAGGCAUGUG | Translation |
|  | gra-miR477 | Gorai.012G160400 | 1 | 21 | 829 | 849 | CGAAGUCUUGGAAGAGAGUAA | AGAUUCUUCUGCAAAACUUUG | Translation |
|  | gra-miR482 | Gorai.004G274000 | 1 | 22 | 270 | 291 | UCUUUCCAAUUCCUCCCAUUCC | UAUGCGAGAGGAAUUGGGGGGA | Cleavage |
|  | gra-miR7486i | Gorai.009G010100 | 1 | 23 | 80 | 102 | GCUGACGUGGAAGGAAAUCGCUA | AACCCCUUUUCUUCUACGUUAAC | Cleavage |
|  | gra-miR7492e | Gorai.002G206900 | 1 | 21 | 531 | 551 | CCAUGAUCUUUAGCGGCGUUU | GGAUGGCGGUCGAGGUUAUGG | Translation |
|  | gra-miR7492f | Gorai.002G206900 | 1 | 21 | 531 | 551 | CCAUGAUCUUUAGCGGCGUUU | GGAUGGCGGUCGAGGUUAUGG | Translation |
|  | gra-miR7492o | Gorai.007G278700 | 1 | 24 | 210 | 233 | CUAAAGAUCUGAGCAUUAGUGGCG | UGUCACUAUUGCUGAGAUCUUGAA | Translation |
|  | gra-miR7502b | Gorai.007G063100 | 1 | 21 | 156 | 176 | UUGUUAAAAGUUUCAUCCAUU | AAUGGGAAGAGCUAUUAAUAA | Cleavage |
|  | gra-miR7502c | Gorai.007G063100 | 1 | 21 | 156 | 176 | UUGUUAAAAGUUUCAUCCAUU | AAUGGGAAGAGCUAUUAAUAA | Cleavage |
|  | gra-miR7502f | Gorai.005G046900 | 1 | 21 | 155 | 174 | UUUAGCAGUAGAAAUAGAUGA | CCAUC-AUUUCUCCUGCGAAA | Translation |
|  | gra-miR7504d | Gorai.012G160400 | 1 | 24 | 860 | 883 | AGGAAAAAAAAUCUGAUUUGUCAU | ACAAAAAAUCAUGUCCUUUUUCUA | Translation |
|  | gra-miR827b | Gorai.008G100300 | 1 | 21 | 34 | 54 | UUUGUUUAUGGUCAUCUAAGC | GCAGAGAUUCCCAUAAACGAA | Cleavage |
|  | gra-miR8641 | Gorai.009G018500 | 1 | 24 | 66 | 89 | UUUUUACUUUGGGACACUGAUGGC | GAUUCAAGUUUCCCAUAGCAAAAA | Translation |
|  | gra-miR8653b | Gorai.008G100300 | 1 | 21 | 400 | 420 | UUCAAACUUAUUUUACGGCCA | AAACCCUUAAAUGAAUUUGAA | Cleavage |
|  | gra-miR8657a | Gorai.004G274000 | 1 | 24 | 250 | 273 | UGUAGUAAUUGUAGAAGUUCAGGG | AUUAUGACUUCUACCAUUGAUAUG | Translation |
|  | gra-miR8657b | Gorai.004G274000 | 1 | 24 | 250 | 273 | UGUAGUAAUUGUAGAAGUUCAGGG | AUUAUGACUUCUACCAUUGAUAUG | Translation |
|  | gra-miR8657c | Gorai.004G274000 | 1 | 24 | 250 | 273 | UGUAGUAAUUGUAGAAGUUCAGGG | AUUAUGACUUCUACCAUUGAUAUG | Translation |
|  | gra-miR8657d | Gorai.004G274000 | 1 | 24 | 250 | 273 | UGUAGUAAUUGUAGAAGUUCAGGG | AUUAUGACUUCUACCAUUGAUAUG | Translation |
|  | gra-miR8657e | Gorai.004G274000 | 1 | 24 | 250 | 273 | UGUAGUAAUUGUAGAAGUUCAGGG | AUUAUGACUUCUACCAUUGAUAUG | Translation |
|  | gra-miR8659a | Gorai.010G064500 | 1 | 24 | 315 | 338 | AAUUUUUUAAGGUUGUUUGUGGAA | GAAGACAGAAAACUUUGAUGAAUU | Cleavage |
|  | gra-miR8659b | Gorai.010G064500 | 1 | 24 | 315 | 338 | AAUUUUUUAAGGUUGUUUGUGGAA | GAAGACAGAAAACUUUGAUGAAUU | Cleavage |
|  | gra-miR8661 | Gorai.007G063100 | 1 | 21 | 407 | 427 | CAUUACUUUUUCAUUCAUUAA | UAAAUGAAUACGAAGAUGAUG | Translation |
|  | gra-miR8665 | Gorai.012G160400 | 1 | 24 | 180 | 203 | UUAAUUAUUAUAUAGAUCAAGGAU | UGUCAUGAUUGCUGAAUUAAUUAA | Translation |
|  | gra-miR8666 | Gorai.008G100300 | 1 | 21 | 309 | 329 | UUGGUGAUCAUAAGUACAUAA | UCAUGUAUCGAUGAUCACUAU | Cleavage |
|  | gra-miR8672 | Gorai.007G063100 | 1 | 24 | 217 | 240 | AAGAGAAAUGAUUGUAUGAAACAG | GAUCUUCAUCAAAUUACUUCUAUU | Cleavage |
|  | gra-miR8694a | Gorai.012G160400 | 1 | 24 | 644 | 667 | AGGAUGCACUGUCAGCAAAAGUAU | AGCCCUUUGCUUUCAAUGUGUUCU | Translation |
|  | gra-miR8694b | Gorai.012G160400 | 1 | 24 | 644 | 667 | AGGAUGCACUGUCAGCAAAAGUAU | AGCCCUUUGCUUUCAAUGUGUUCU | Translation |
|  | gra-miR8703a | Gorai.007G063100 | 1 | 24 | 88 | 111 | AGUAGUCUAAUUGGUAUAGCUGAA | AGGAAUUAUAUUACUUAUGCUACU | Translation |
|  | gra-miR8703b | Gorai.007G063100 | 1 | 24 | 88 | 111 | AGUAGUCUAAUUGGUAUAGCUGAA | AGGAAUUAUAUUACUUAUGCUACU | Translation |
|  | gra-miR8703c | Gorai.007G063100 | 1 | 24 | 88 | 111 | AGUAGUCUAAUUGGUAUAGCUGAA | AGGAAUUAUAUUACUUAUGCUACU | Translation |
|  | gra-miR8705 | Gorai.012G160400 | 1 | 23 | 40 | 62 | AGUUCAUACUAGUUCGUGGGUCA | ACACCCAUAAACGAGAAUGAACU | Translation |
|  | gra-miR8708 | Gorai.004G058400 | 1 | 23 | 97 | 120 | AGGAGGAGUUAUGG-AUAGUUUUA | AUAAACUAUGCCAUUGCUCUUCUU | Translation |
|  | gra-miR8717 | Gorai.008G100300 | 1 | 24 | 229 | 252 | AUUGGUUGUUCUGAUUCGAGGCUA | AAUACUUCAAUUGGAUCAACUGAU | Translation |
|  | gra-miR8722 | Gorai.002G206900 | 1 | 22 | 463 | 484 | CAUGUUUUUCCUGUUCAUCUUC | CAUGUUGGACAAGGAAAAUAUG | Translation |
|  | gra-miR8723b | Gorai.002G140000 | 1 | 24 | 281 | 303 | CCAUUAACGGUGUAACAGUAAGCU | ACACAACUGUUGCAAC-UUCAUGG | Translation |
|  | gra-miR8732 | Gorai.012G160400 | 1 | 24 | 814 | 837 | GAAGAGUAUAGGGACUUAUGGCAU | AGAGCAUUAAUCCUUAGAUUCUUC | Cleavage |
|  | gra-miR8741 | Gorai.009G018500 | 1 | 21 | 26 | 46 | UAGCACUGAAGAUGAUGAUGG | ACAACAUCAACUUUGGCGCUG | Cleavage |
|  | gra-miR8742a | Gorai.007G063100 | 1 | 21 | 605 | 625 | UAUCUUAUUCAUCUUGGACUG | GCGUCUAUGCUGAAUCAGAUG | Cleavage |
|  | gra-miR8742b | Gorai.007G063100 | 1 | 21 | 605 | 625 | UAUCUUAUUCAUCUUGGACUG | GCGUCUAUGCUGAAUCAGAUG | Cleavage |
|  | gra-miR8752 | Gorai.002G047400 | 1 | 21 | 364 | 384 | UGAUGGAGAUAGGUAUCUGCA | GCCGGGUAUCAACCUCCAUUA | Translation |
|  | gra-miR8764 | Gorai.007G063100 | 1 | 21 | 486 | 506 | UUAGAUUGCAUUUUACCCCUU | UAGGGCUAAAGGGGAAUAUAA | Translation |
|  | gra-miR8767c | Gorai.005G046900 | 1 | 21 | 483 | 503 | UUUUCAACUCUGCCAAGCAAU | UGUGCUGGGAAAAGCUGAGAA | Translation |
|  | gra-miR8768 | Gorai.012G160400 | 1 | 21 | 249 | 269 | UUCCAUGUCACAGAGAUGUUG | AGACAUAACUGACACAUGGGA | Translation |
|  | gra-miR8770 | Gorai.012G160400 | 1 | 24 | 91 | 113 | UUGAUGGUGGUAAGAAAUGUGCAU | AAUUAUAUUUCUUAC-GCCAUCAC | Translation |
|  | gra-miR8774 | Gorai.004G058400 | 1 | 24 | 455 | 478 | UUGGAUUUUGAUUCAUAGAUUCGU | AUAAUUCUGUUAAUGAAGAUUCAU | Translation |
|  | gra-miR8775 | Gorai.004G058400 | 1 | 21 | 417 | 437 | UUGUGAGAUUGAAGCUGAUGG | UCAUCAGCAGCAAUCACAGAA | Cleavage |
|  | gra-miR8777 | Gorai.012G160400 | 1 | 20 | 896 | 915 | UUUCCAAUAGAAGAAUGACA | CUUUGUUUUUCACUUGGAGA | Translation |
|  | gra-miR8782 | Gorai.004G058400 | 1 | 21 | 209 | 229 | UUUGGUGUUGAAGGGGAAUAA | GGGUUCCCCAACUGCAUCAAG | Translation |
|  | gra-miR8783 | Gorai.007G063100 | 1 | 21 | 299 | 319 | UUUGUACGUGGCGGGAGAUAU | AGAUUACCCGUCAUGUAUCGA | Cleavage |
|  | gra-miR8785 | Gorai.002G047400 | 1 | 21 | 175 | 195 | UUUUACAGCAGCUACAUCCAU | AAGGCUGUAGCUAUUGUAGAG | Translation |
|  | gra-miR8787 | Gorai.013G105600 | 1 | 23 | 48 | 70 | UUUUCUUUUAAUUGGACGAGAUA | AUCGCCCUCCAACAAAAAGAAGA | Translation |
|  | han-miR3630-3p | Gorai.008G100300 | 1 | 22 | 26 | 48 | UGUGGGAAUCUCU-CUGAUGCUU | AGCCAAAAGCAGAGAUUCCCAUA | Cleavage |
